# Advancements in GelMA bioactive hydrogels: Strategies for infection control and bone tissue regeneration

**DOI:** 10.7150/thno.103725

**Published:** 2025-01-01

**Authors:** Lei Huang, Ziyao Guo, Xiaoxia Yang, Yinchun Zhang, Yiyun Liang, Xiaxue Chen, Xiaoling Qiu, Xuan Chen

**Affiliations:** 1 Department of Endodontics, Stomatological Hospital, School of Stomatology, Southern Medical University, Guangzhou, China.; 2 SCP 11A of the International Department, Guangzhou Experimental Foreign Language School, Guangzhou, China.; 3 Department of Periodontology, Shaoxing Stomatological Hospital, Shaoxing, Zhejiang, China.

**Keywords:** GelMA hydrogels, infection control, bone tissue regeneration, infected bone tissue regeneration, antimicrobial materials

## Abstract

Infectious bone defects present a significant clinical challenge, characterized by infection, inflammation, and subsequent bone tissue destruction. Traditional treatments, including antibiotic therapy, surgical debridement, and bone grafting, often fail to address these defects effectively. However, recent advancements in biomaterials research have introduced innovative solutions for managing infectious bone defects. GelMA, a three-dimensional network of hydrophilic polymers that can absorb and retain substantial amounts of water, has attracted considerable attention in the fields of materials science and biomedical engineering. Its distinctive properties, such as biocompatibility, responsiveness to stimuli, and customisable mechanical characteristics make GelMA an exemplary scaffold material for bone tissue engineering. This review aims to thoroughly explore the current literature on antibacterial and osteogenic strategies using GelMA hydrogels for the restoration of infected bones. It discusses their fabrication methods, biocompatibility, antibacterial effectiveness, and bioactivity. We conclude by discussing the existing challenges and future research directions in this field, with the hope of inspiring further innovations in the synthesis, modification, and application of GelMA-based hydrogels for infection control and bone tissue regeneration.

## Introduction

Infectious bone defects present a formidable clinical challenge due to the immunological response to microbial invasion and the release of acidic metabolites. Collectively, these factors hinder osteoblastic activity, thereby obstructing the natural healing of bone defects. Such conditions are prevalent among individuals with trauma, surgical history, or diabetes, significantly affecting their quality of life 1. Traditional treatments, including bone grafting and antibiotic therapy, are effective but limited by issues such as restricted bone supply and the emergence of secondary diseases 2,3. Furthermore, the escalating problem of antibiotic resistance poses a considerable public health threat 4,5. Consequently, therapeutic strategies for infectious bone defects must balance the control of antimicrobial inflammation with the promotion of bone healing.

The advent of tissue-engineering technologies has introduced novel treatment approaches in this field. By integrating biodegradable scaffolds, seed cells, and cytokines, tissue engineering offers a biological alternative for repairing infectious bone defects and provides new perspectives and methods for clinical treatment. The repair process of bone defects is both orderly and dynamic, involving multiple dimensions. The selection of an appropriate scaffold substitute is crucial for this process. With the advancements in biomedical materials, hydrogels have garnered significant attention. Hydrogels are highly hydrophilic three-dimensional networks that serve as biological scaffolds for cell growth 6,7. They rapidly expand in water, retain a significant amount of moisture without dissolving, and resemble the extracellular matrix (ECM), providing structural support for bone defect sites and promoting healing through intrinsic mechanisms 6. Hydrogels are simple to prepare, cost-effective, and exhibit low toxicity, making them widely used in bone tissue engineering. By mimicking the ECM environment, hydrogels provide an ideal biological scaffold for the repair of bone defects and facilitate the regeneration and healing of bone tissue 8,9.

GelMA hydrogels, which are biomaterials based on gelatine, can form crosslinked three-dimensional networks under specific conditions through methacrylation modification 10. Gelatine, a natural protein derived from collagen, is a macromolecule obtained from animal skin, bone, and connective tissue, and possesses excellent biocompatibility and degradability 11. The methacrylation modification endows GelMA hydrogels with improved mechanical properties and processability while retaining the biological features of gelatine 12. GelMA hydrogels possess the versatility to amalgamate with cells, growth factors, pharmaceuticals, or exosomes, fostering microenvironments conducive to osteogenesis, angiogenesis, and antimicrobial efficacy 10. Therefore, GelMA hydrogels are widely employed in drug delivery, tissue engineering, biological research, and hydrogel devices 12-14. An ideal bone regeneration scaffold should possess excellent cellular/biocompatibility, outstanding bioactivity, biodegradability, suitable biomechanical properties, and necessary porous structures to facilitate the adhesion, proliferation, diffusion, nutrient transport, and gas exchange of osteoblasts 11,15. However, pure GelMA hydrogels exhibit inadequate osteogenic activity. Therefore, to enhance the osteogenic capacity of the composite materials, it is essential to combine GelMA hydrogels with materials that possess osteogenic properties. Additionally, the mechanical strength of GelMA hydrogels is relatively weak, and their electrical conductivity is insufficient, making the development of hybrid hydrogels composed of two or more components a crucial strategy 16. This approach leverages the unique advantages of each component and provides promising possibilities for developing tissue-engineered biomaterials. Notably, the concentration and degree of substitution of GelMA significantly influence its physicochemical properties and cellular responses, leading to a degree of uncertainty and limitation in bioactivity modulation. In bone defect repair applications, the poor mechanical stiffness and uncontrollable degradation rate of pure GelMA hydrogels severely restrict their widespread use in load-bearing bone defect treatments. Thus, multifunctional bone tissue engineering scaffolds with osteogenic and antibacterial properties can be produced by employing various strategies to modify pure GelMA hydrogels and incorporate bioactive components 15.

**Scheme 1:**
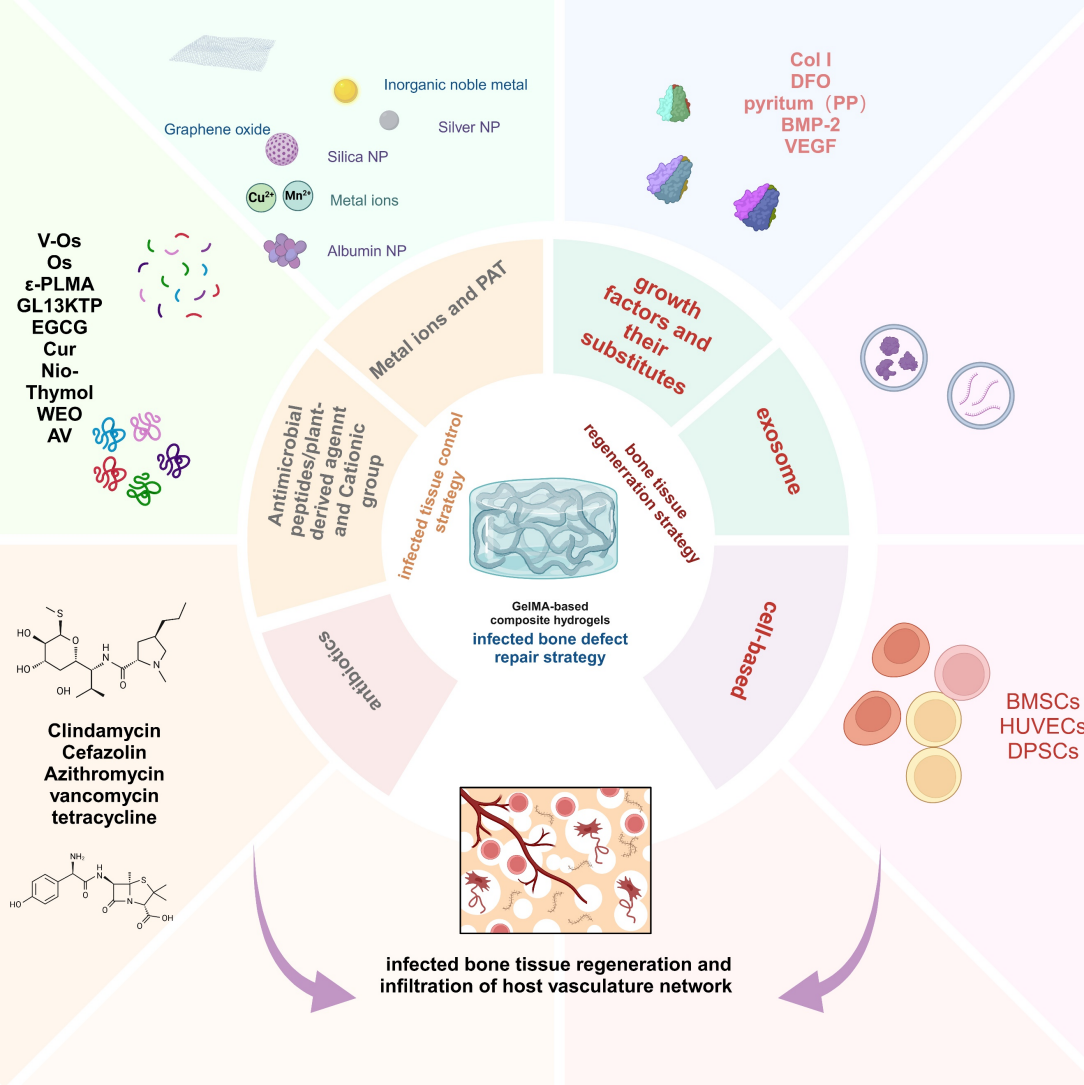
Schematic description of GelMA-based composite hydrogels with multiple functions for infection control and bone tissue regeneration (created with BioRender.com/y41f851).

This review provides a comprehensive synopsis of the use of GelMA hydrogels for infection control and bone tissue regeneration. This section discusses the composition and structure of the GelMA hydrogels and explores their multifunctional properties. Special focus is given to the advancement of GelMA hydrogels tailored for the resolution of infectious bone defects, particularly those incorporating infection-mitigation agents such as antibiotics, metallic ions, antimicrobial peptides (AMPs), botanical active compounds, photothermal therapy (PTT), and intelligent responsive mechanisms (Scheme 1). This review provides a comprehensive overview of the incorporation of various antimicrobial agents into GelMA hydrogels for bone repair and their antimicrobial attributes, thereby providing a theoretical basis for the advancement of GelMA hydrogels in the treatment of infectious bone defects. Furthermore, it addresses the inherent challenges in the application of GelMA hydrogels. Finally, recent progress and technical directions for GelMA hydrogels are discussed, with the aim of promoting the development of novel composites based on GelMA hydrogels and expanding their future application prospects.

## Hydrogel overview

### Properties of hydrogels

Hydrogels are three-dimensional crosslinked porous materials characterised by excellent permeability, remarkable drug-carrying capacity, high water content, optimal mechanical strength, favourable biocompatibility, and sensitivity to environmental stimuli such as temperature, pH, and solvent type. The polymer network of hydrogels is composed of chains linked at crosslinking points, forming a three-dimensional structure whose stability and functionality are influenced by the polymer type, degree of crosslinking, and external physicochemical conditions. The polymer type primarily affects the chemical properties and biocompatibility of the hydrogel, whereas the degree of crosslinking dictates the structural density, influencing its water absorption capacity and mechanical stability. Hydrogels possess a 3D structure that closely mimics the ECM, supports the activity of biomolecules and cells, and offers an ideal platform for cell adhesion, proliferation, and migration at wound sites 17. Furthermore, their exceptional water absorption and swelling properties allow them to absorb excess exudate without dissolving, while the pore size can be tailored to encapsulate various cells, drugs, growth factors, nucleic acids, nanoparticles, metal ions (e.g., Mg^2+^, Fe^3+^), gas molecules (e.g., NO, O₂), and therapeutic agents like platelet-rich plasma (PRP) 18,19. Therefore, hydrogels are widely regarded as promising materials for bone tissue engineering applications.

### Classification of hydrogels

Natural polymer hydrogels are composed primarily of proteins, peptides, polysaccharides, and nucleic acids. Hydrogels based on proteins or peptides mainly include gelatine, collagen, fibrin, silk fibroin, and ε-polylysine (EPL), which retain a higher concentration of cell-adhesive proteins and exhibit excellent biocompatibility 20. Polysaccharide-based hydrogels comprise chitosan, alginates, agarose, hyaluronic acid, glucans, cellulose, guar gum, and cyclodextrin polymers. Natural polymer hydrogels offer numerous advantages, including abundant sources, porous structures, multifunctional groups, favourable swelling properties, biodegradability, and low immunogenicity. However, the mechanical properties of these materials tend to be inferior. To enhance their physicochemical characteristics, chemical modifications can be used to introduce additional biologically functional sites 21. A notable example is the methacrylation of hydrogels, which imparts remarkable photosensitivity 22. Furthermore, the quaternization of chitosan hydrogels enhances their antibacterial properties, whereas catechol modification imparts exceptional tissue adhesion capabilities 23. Synthetic hydrogels are derived primarily from organic polymers. The commonly synthesised hydrogels include polyacrylamide (PAM), polyethylene glycol (PEG), polyvinyl alcohol (PVA), polyurethane (PU), polyvinylpyrrolidone (PVP), polyethylene imine (PEI), poly(N-isopropylacrylamide) (PNIPAM), Pluronic F127 (PF127), polylactide-co-glycolide (PLGA), polyhydroxyalkanoates (PHA), polylactic acid (PLA), polycaprolactone (PCL), polymethacrylic acid (PMAA), and their derivatives. These synthetic hydrogels are valuable owing to their stability, structural strength, resistance to degradation, and potential for mass production. However, they may raise certain biological safety concerns due to the possible release of monomers and toxic substances during synthesis 18.

Both hydrogel categories can be combined to create hybrid hydrogels. For example, polyvinyl alcohol crosslinked with sodium alginate (PVA-SA) hybrid hydrogels exhibit good biocompatibility and mechanical properties, making them suitable for the localised delivery of phages and antibiotics for the treatment of methicillin-resistant *Staphylococcus aureus* infections in burn wounds 24. Additionally, polyvinylpyrrolidone crosslinked with hyaluronic acid (PVP-HA) enhanced the mechanical strength of the microneedles, ensuring effective drug permeation. Moreover, the incorporation of nanomaterials into hydrogels endows them with stimulus-responsive characteristics 25 (Table 1).

## Characterization and preparation of GelMA hydrogel

GelMA, short for gelatine methacrylate, was initially synthesized by Van Den Bulcke and colleagues in 2000 35. Derived from collagen hydrolysis, this material inherits the desirable solubility and low antigenicity of gelatine. Notably, the retention of the arginine-glycine-aspartate (RGD) sequence is a critical structure that facilitates cell adhesion, proliferation, and differentiation 8. Moreover, the presence of matrix metalloproteinases (MMPs) within gelatine aids cell remodelling, thereby enhancing the physicochemical properties of the material 36.

GelMA is prominent in the biomedical field because of its remarkable versatility. The extensive applications of GelMA hydrogels can be largely attributed to their unique biological properties, which facilitate superior cell attachment and proliferation across a wide array of cell types. In summary, there are four primary reasons that researchers opt for gelatine methacrylate:

I. Biocompatibility: GelMA gelatine molecules feature RGD sequences that promote the adhesion and proliferation of virtually any cell type 37. These sequences, derived from collagen, are retained in the material to support cell adhesion, proliferation, and maturation within the construct.

II. Biodegradability: GelMA contains MMP sites that enable its enzymatic degradation by cells. As natural cells populate a construct, they progressively degrade and remodel the material, ultimately replacing the original structure with their own cells and tissues 22.

III. Tunable properties: GelMA possesses excellent tunable properties, allowing easy customisation of the hydrogels. This flexibility arises from the degree of substitution in GelMA, which directly influences the stiffness of the polymer and its mechanical properties, including compression and tensile strength. By adjusting these parameters, the soft and hard components of natural tissues can be mimicked, thereby activating biomechanical signals in specific microenvironments and guiding the maturation of embedded cells in designated orientations 10.

IV. Bioprintability: GelMA exhibits outstanding bioprintability. It is extensively utilised in both research and commercial applications because of its capacity to create complex structures with diverse characteristics. Scientists have employed GelMA to fabricate intricate 3D structures using bioprinting technology, providing cells with growth conditions similar to those in the body 10,38.

Recently, significant advances have been made in bone tissue engineering for the design and fabrication of artificial bone scaffolds. Scaffolds can be classified into two main categories: natural and synthetic. Common synthetic materials include polylactic acid, polyglycolic acid, and inorganic compounds such as titanium alloys, calcium phosphate, bioceramics, and bioactive glass 39. The incorporation of inorganic minerals, particularly calcium phosphate, has demonstrated beneficial effects on bone regeneration, owing to their biocompatibility and osteoconductivity 16. However, the practical application of these materials is challenging because of their fragility and limited adaptability 16. Conversely, synthetic organic materials, such as polylactic acid, can be easily fabricated with desirable mechanical properties and acceptable biocompatibility; however, they often lack the necessary bioactivity to initiate bone repair.

Consequently, researchers have shifted their focus toward natural materials as potential sources for developing bone regeneration scaffolds with high biocompatibility and low cytotoxicity. Many researchers have integrated GelMA into bone repair material systems owing to its favourable temperature-sensitive gel properties, degradability, adjustable mechanical characteristics, and ability to promote bone differentiation and vascularisation 16,40.

### Synthesis of GelMA hydrogel

Since its advent in the 21st century, GelMA has garnered widespread application in numerous subfields of tissue engineering, including antibacterial, osteogenic, drug delivery, and gene transfer applications. Over the past two decades, researchers have reported various methods for synthesising GelMA, each with its own merits but fundamentally rooted in the general synthetic route initially proposed by Bulcke and colleagues 35, albeit with varying degrees of optimisation and adjustment within this framework. A concise overview of the GelMA synthesis process is provided below.

Typically, GelMA is synthesised in phosphate buffer solution (pH 7.4) through the direct reaction of gelatine with methacrylic anhydride (MA). In this process, methacrylate substituents are introduced into the amino and hydroxyl groups of amino acid residues 41. By controlling the amount of MA added to the reaction mixture, GelMAs with varying degrees of methacrylate substitution can be synthesised, resulting in materials with diverse physical properties. Maintaining the alkalinity of the reaction solution is crucial for augmenting the reactivity of the amino and hydroxyl groups and ensuring a higher level of substitution. In the final stage, the substitution reaction is halted by adding a five-fold diluted phosphate buffer solution to the reaction mixture 42. Subsequently, the mixture is dialysed against deionised water using a dialysis membrane with a molecular weight cutoff of 12-14 kDa for 5-7 days to eliminate low-molecular-weight impurities that may affect cell function (e.g., unreacted MA and methacrylic acid byproducts). If required, GelMA can be preserved by freeze-drying and reconstituted for use at room temperature.

### Tunability of GelMA mechanical properties

The tunability of the mechanical properties of GelMA is a defining feature of the material, which significantly contributes to its sustained interest in tissue engineering applications. By adjusting the degree of methacrylate substitution, the pore size of the GelMA hydrogels can be precisely controlled, which is a critical factor in facilitating cellular nutrient exchange. Studies have demonstrated that different concentrations of MA (1M, 5M, and 10M) can yield GelMA hydrogels with varying degrees of substitution (49.8%, 63.8%, and 73.2%, respectively). Following freeze-drying, the average pore size of these hydrogels is reported to have decreased from 49.7±11.8 μm for 1M to 23.6±5.85 μm for 10M, as confirmed by FE-SEM, indicating a decrease in pore size with increasing substitution degree 43. This observation has been corroborated by other experiments, which revealed an inverse relationship between the degree of methacrylate substitution and the mass fraction of GelMA 43. Although GelMA with a high degree of substitution exhibits greater mechanical strength, it hinders cell proliferation and is therefore unsuitable for cell loading and co-culture. Some researchers have conducted two-dimensional nuclear magnetic resonance (NMR) experiments to obtain detailed information on the signals present in the 1H NMR spectrum. Using this method, both methacrylamide from the methacrylate groups and unreacted impurities, such as methacrylic acid, can be identified. This quantitative cationic approach enables precise control over the substitution of GelMA, allowing for further adjustments to the hydrogel properties to meet the requirements of bone regeneration scaffolds 44. Additionally, studies have shown that a higher degree of methacrylation in GelMA is correlated with a lower viscosity of the GelMA precursor aqueous solution, making it suitable for 3D bioprinting 18.

Despite possessing osteogenic properties, such as cell adhesion, proliferation, and differentiation, the mechanical properties of GelMA hydrogels are insufficient for use as load-bearing materials. Wang *et al.* 45 devised a novel two-component polymer hydrogel, GelMA-DexMA, via photopolymerization using GelMA and dextran glycidyl methacrylate (DexMA). They reported that improved mechanical properties can be attained by regulating the degree of substitution (DS) of glycidyl methacrylate in DexMA. GelMA-DexMA exhibits a honeycomb-like structure with decreasing pore size as the DS increases, displaying a lower tensile strength and higher compressive modulus. The UV dose used during polymerisation also plays a pivotal role in determining the mechanical properties of GelMA. Substituted gelatine derivatives are highly prone to photoinduced reactions because of the abundance of unsaturated photocrosslinking groups. The primary amine (-NH₂) and hydroxyl (-OH) groups predominantly participate in this substitution reaction, facilitating the introduction of methacryloyl groups into gelatine. In the presence of a photoinitiator, GelMA polymerisation occurs in an aqueous solution via a free-radical mechanism. UV irradiation of the photoinitiator generates free radicals through homolytic cleavage, which initiates chain polymerisation 46. Photopolymerization offers numerous advantages over other methods, including injectability, rapid gelation, enhanced mechanical properties, compatibility with custom bioprinting, and ease of integration into various cell types 22,47. However, free radicals produced during crosslinking can attack cell membranes, resulting in cell death 48,49. This detrimental effect is contingent upon the radiation source and intensity of the UV light. Research has also demonstrated that photocrosslinking can be biocompatible under mild conditions and can be easily adjusted by reducing the intensity of the UV source and the amount of the photoinitiator used. Commonly employed photoinitiators are free radical photoinitiators, such as LAP, IC-2959, and VA086, which exhibit good biocompatibility and low immunogenicity 46. Wang *et al.* 50 synthesised GelMA/polyethylene glycol diacrylate (PEGDA) gels using the UV photocrosslinking method by modifying photoinitiators I and II. They reported that the gelling time could be readily controlled by altering the amounts of I2959 and PEGDA photoinducers. It was also observed that the mechanical strength, spreading, and swelling rates of this type of gel were enhanced, with mechanical properties significantly superior to those of GelMA. Although LAP initiation proved to be more efficient, yielding hydrogels with higher moduli than those initiated by IC-2959 and VA086 under identical curing conditions, the GelMA hydrogel could also be chemically crosslinked using agents such as glutaraldehyde. However, because of its high toxicity to living organisms, glutaraldehyde is typically unsuitable for cell culture applications 51,52. Furthermore, different synthesis systems can influence the structure and properties of GelMA. Currently, the mainstream method for GelMA synthesis involves a phosphate buffer solution (PBS) reaction system. Several research groups have reported alternative methods for synthesising GelMA. For instance, recent studies have indicated that GelMA synthesised in a carbonate-bicarbonate buffer solution (CBS) exhibits superior deprotonation of free amino groups and buffering capacity compared with GelMA synthesised in PBS 11,53. Additionally, the synthesis of GelMA using the CBS reaction system requires a reduced amount of MA, rendering the synthesis both environmentally friendly and cost-effective 11,53. In addition to optimising the degree of substitution and photopolymerization time, GelMA can be combined with other materials to achieve enhanced performance. Wang *et al.* 54 enhanced the mechanical properties of GelMA hydrogels by simple physical mixing with nanoscale hydroxyapatite nanoparticles. Pressure testing revealed that addition of 5% nanoscale hydroxyapatite results in the hydrogel withstanding a maximum compressive stress of (278.62±7.49) kPa, over three times higher than GelMA hydrogels without nanoparticles, with no significant change in pore size. It is also observed that co-modification of GelMA with type I collagen, a natural polymer, notably improves the rheological properties of the hydrogel, such as viscosity and stiffness, and enhances the viability of human umbilical vein endothelial cells (HUVECs) 55. Zuo *et al.* 56 developed a novel system comprising GelMA and HA scaffolds loaded with HUVEC and human osteosarcoma-like cells (MG63). Compared with pure GelMA hydrogels, these composite hydrogels exhibit lower swelling, higher mechanical strength, and superior biocompatibility. In this age of innovative materials, the exceptional tunability of GelMA sets it apart and injects renewed vitality into the field.

## Bone defect repair strategy

In the field of bone tissue engineering, strategies for the reconstruction of bone defects using GelMA hydrogels have been extensively explored 15, and a succinct overview of these strategies is presented in Table 2, focusing on the dual functionality of GelMA hydrogels in infection control and antibacterial osteogenesis for infectious bone defects. Infectious bone tissue defects often pose challenges to healing via intrinsic bone repair mechanisms, necessitating biomimetic reconstruction techniques to restore bone shape and function. This typically involves the use of materials from the field of bone tissue engineering 87. Ideal candidates for bone regeneration should exhibit biomechanical strength, physicochemical properties, optimal porosity, biodegradability, biocompatibility, and osteoconductivity, while serving as conduits for the unhindered transport of nutrients, waste, and gases for cells embedded within the scaffold 1,4. Integrated bone-graft materials should possess at least one of the following biological attributes: 1. osteoconductivity, providing a framework for tissue ingrowth and new bone deposition; 2. osteoinductivity, secreting factors that stimulate the differentiation of osteoprogenitor cells into bone; and 3. osteogenicity, supplying bone progenitor cells with osteogenic potential 15. These criteria can be met by employing GelMA scaffolds as primary vehicles for the delivery of drugs, cells, growth factors, and exosomes, thereby enhancing the repair process. Consequently, the development and design of GelMA-based drug delivery systems can achieve local delivery, ensuring that the effective concentration is maintained for a sustained duration, thereby maximising the therapeutic efficacy.

### Stem cell

The repair of bone defects is a multifaceted process involving two fundamental multicellular units: osteoclasts and osteoblasts 88. Cell-based therapies have emerged as effective strategies for bone repair. Stem cell therapy typically involves injecting a suspension of stem cells into the site of the bone defect. However, the microenvironment within the defect presents challenges in maintaining the quantity and vitality of stem cells 89. Therefore, hydrogels containing stem cells should be implanted into bone defects to create a cellular microenvironment conducive to adhesion, proliferation, and differentiation 89.

Bone marrow mesenchymal stem cells (BMSCs) can self-renew and differentiate into multiple lineages, making them the ideal seed cells for bone tissue engineering 90. These cells secrete paracrine mediators and nutrients, regulate immunity, promote angiogenesis, and exhibit excellent osteogenic properties 91. The hydrogel scaffolds prepared with bio-GelMA and encapsulated BMSCs demonstrate good biocompatibility and are chemically and physically similar to the natural extracellular matrix of bone cells. As a result of loading the hydrogel scaffold with BMSCs, new bone has been reported to form most effectively in rat segmental bone defect models 57.

Researchers have devised injectable hydrogels to address the clinical need for irregular bone defects. Shi *et al.* 58 used a bone-mimicking and injectable GelMA hydrogel (GelMA-HAP-SN) system containing mesenchymal stem cells (MSC), nano-hydroxyapatite (HAP), and nano-silicate (SN) for bone tissue engineering and systematically examined the osteogenic capacity of GelMA-HAP-SN *in vitro* and *in vivo*. The incorporation of HAP enhanced the resemblance to the components of the natural extracellular matrix of bone cells, whereas SN loading conferred injectability and osteogenic properties to the hydrogel. Consequently, GelMA-HAP-SN hydrogels exhibited increased cell viability, proliferation, and diffusion. Additionally, the GelMA-HAP-SN hydrogels augmented the expression of osteogenic biomarkers and matrix mineralisation in the encapsulated MSCs. Moreover, injection of MSC-encapsulated GelMA-HAP-SN hydrogels into critical-sized cranial bone defects in rats further confirmed their outstanding bone regeneration capacity.

**Figure 1:**
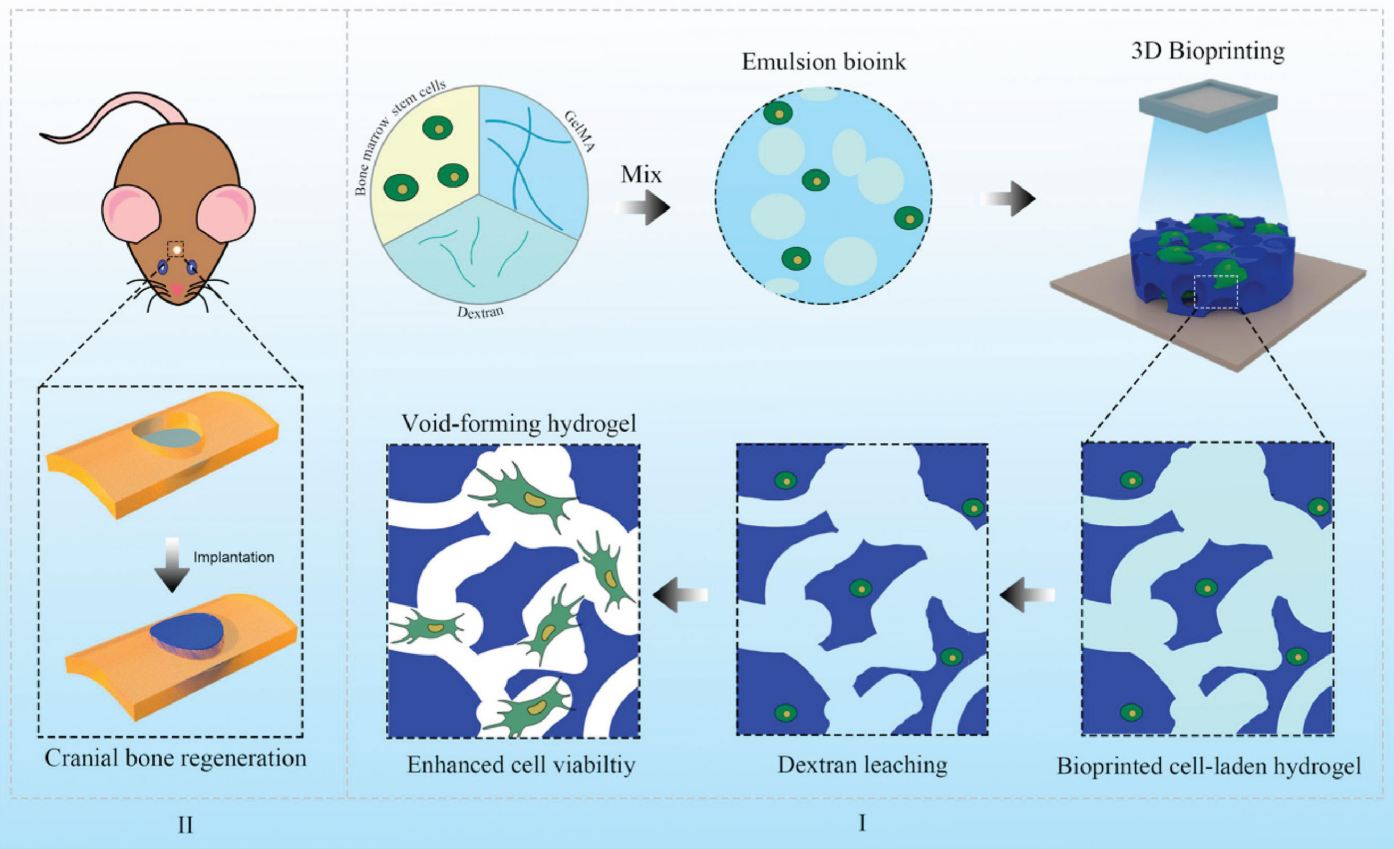
Schematic illustration of the 3D-bioprinted void-forming hydrogel constructs for implantation. (A) An aqueous emulsion bioink, where dextran solution is distributed in droplet form within a GelMA solution, was prepared by mixing GelMA, dextran, and stem cells for 3D bioprinting of a void-forming hydrogel to (B) repair the cranial defect. Reproduced from reference 61. Copyright 2022, with permission from Elsevier.

Technological advances have facilitated the application of GelMA to irregular bone defects. Tao *et al.* 59 have integrated 3D bioprinting with stem cells to engineer cell-laden hydrogels for bone regeneration. This hydrogel was formulated by bioprinting BMSCs with a GelMA/dextran emulsion via digital light processing (DLP). 3D bioprinted hydrogels not only foster the migration, proliferation, and diffusion of encapsulated BMSCs but also stimulate the YAP signalling pathway, thereby enhancing osteogenic differentiation of BMSCs. Furthermore, *in vivo* therapeutic evaluations have indicated that hydrogels with pores exhibit significant potential for BMSC delivery, effectively promoting bone regeneration (Figure 1).

### Growth factors and their substitutes

Growth factors are a class of peptides that facilitate communication between cells and regulate cell growth, forming the fundamental triad of biotechnological tissue engineering alongside seed cells and scaffold materials 92. These factors play crucial roles in the promotion of cell proliferation, tissue repair, and organ regeneration 93. In the realm of tissue engineering research, a myriad of growth factors has been recognised and new discoveries are continually emerging. Given the significant reliance of the bone healing process on biologically active factors, exogenous biologically active agents can be introduced via injection into traditional methods to expedite bone repair. However, it is imperative to acknowledge that most biologically active agents are proteins, which are susceptible to enzymatic degradation 94. Therefore, appropriate carriers must be employed to ensure safe delivery of active agents and prevent their inactivation. Precise delivery of growth factors and minimisation of rapid diffusion are also important to avoid potential inflammatory reactions and other adverse effects 95. GelMA hydrogels possess a favourable three-dimensional structure, adjustable swelling, and porosity, enabling the controlled release of biologically active factors in both spatial and temporal dimensions, thereby maintaining effective concentrations over extended periods 96. In the context of bone regeneration, bone morphogenetic proteins (BMPs) 97, vascular endothelial growth factor (VEGF) 98, insulin-like growth factor (IGF) 99, and fibroblast growth factor (FGF) 100 stand as the most extensively studied and utilized growth factors. Additionally, certain pharmaceuticals and hormones, such as dexamethasone, exhibit osteogenic effects and can serve as alternatives to growth factors in bone tissue engineering.

BMP: The BMP family, a subset of the TGF-β superfamily, plays a pivotal role in cell growth, mesenchymal stem cell differentiation, and tissue regeneration and remodelling 101. These factors can induce MSCs to differentiate into various tissues, including bone, cartilage, ligaments, tendons, and nerves. BMP-2 and BMP-7 are commonly used for bone and cartilage regeneration. Calcium carbonate (CaCO3), which has exceptional biocompatibility and bioactivity, is a well-established bone defect-filling material. Lu *et al.* 73 synthesised CaCO3 microspheres (CM) as intelligent carriers to load BMP-2. Subsequently, CM loaded with BMP-2 and catalase (CAT) was incorporated into GelMA hydrogels to fabricate a composite hydrogel for differential drug release. They reported that CAT within the hydrogel was rapidly released to eliminate H2O2 and generate oxygen, whereas the CM continuously released BMP-2 to facilitate rapid osteogenesis. *In vitro* experiments demonstrated that this composite hydrogel effectively reduced intracellular reactive oxygen species levels, thereby preventing cell damage from oxidative stress, enhancing cell survival and proliferation, and strengthening osteogenic properties. Animal experiments further revealed that this composite hydrogel could mitigate inflammation, modulate macrophage polarisation, and promote bone-defect healing.

**Figure 2:**
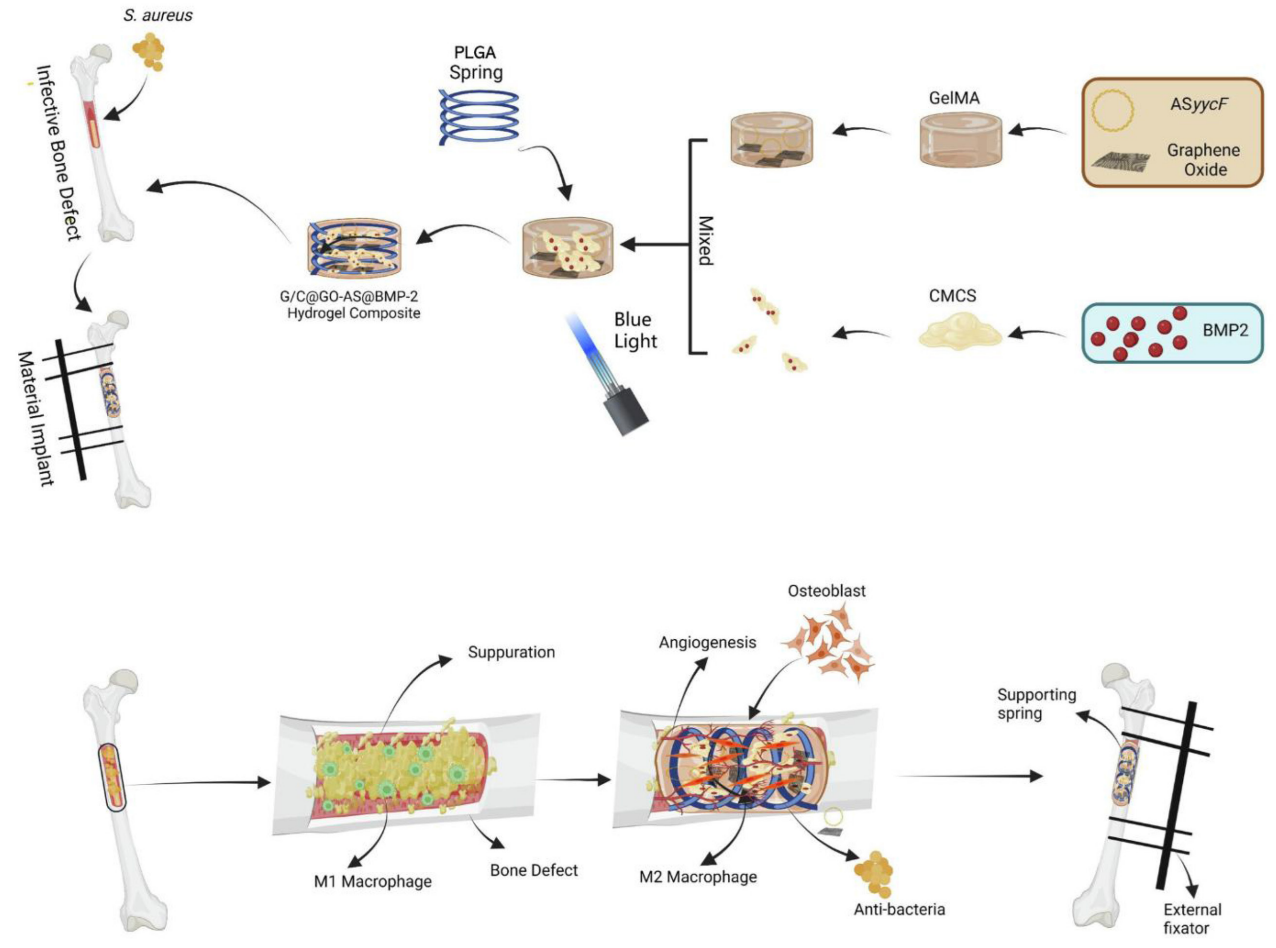
The synthesis of PLAG spring supporting G/CB@GOAS hydrogel composite and application in infected femur defect models. The GelMA precursor solution loaded with the GO-ASyycF system was mixed with the CMCS solution loaded with BMP-2. After application of PLGA spring, the P-G/CB@GOAS hydrogel composite was synthesized by 405 nm blue light photo-crosslinking. The hydrogel composite was implanted into the femur infected bone defects of rats and the external fixator was applied for fixation. BMP-2 mainly induced bone regeneration. GO-ASyycF system mainly played the role of antibacterial. Meanwhile, the hydrogel composite also induced angiogenesis and promoted M2 macrophage polarization. They played synergistic roles in the treatment of infected bone defects. Reproduced from reference 103 Copyright 2023, with permission from Elsevier.

Qin *et al.* 102 developed a GelMA and carboxymethyl chitosan (CMCS) composite hydrogel that incorporates BMP-2 growth factor and a post-transcriptional regulation antisense technique based on graphene oxide (GO). The photocrosslinked GelMA composite hydrogel exhibited excellent biocompatibility and *in vitro* degradation. As the GelMA and CMCS composite hydrogels degraded, antisense yycF and BMP-2 were released. In terms of antibacterial properties, the synergistic action of CMCS, GO, and post-transcriptional regulation antisense yycF in the composite hydrogel effectively eradicated *S. aureus*. In *in vivo* experiments, in which the composite hydrogel was implanted into a rat model with an *S. aureus*-infected femoral defect, bone healing was significantly accelerated in an infectious microenvironment (Figure 2). To address the repair of irregular bone defects, Chai *et al.* 75 developed a photocrosslinked composite biologically active scaffold based on GelMA, BMSCs, and BMP-2. This composite scaffold exhibited suitable mechanical properties for stem cell adhesion and proliferation, good biocompatibility, and the ability to stimulate the osteogenic differentiation of BMSCs *in vitro*. Imaging and histological analyses demonstrated that the synergy between BMSCs and BMP-2 in this composite biologically active scaffold exhibited higher osteogenic potential *in vivo* than scaffolds loaded with BMSCs or BMP-2 alone.

**Figure 3:**
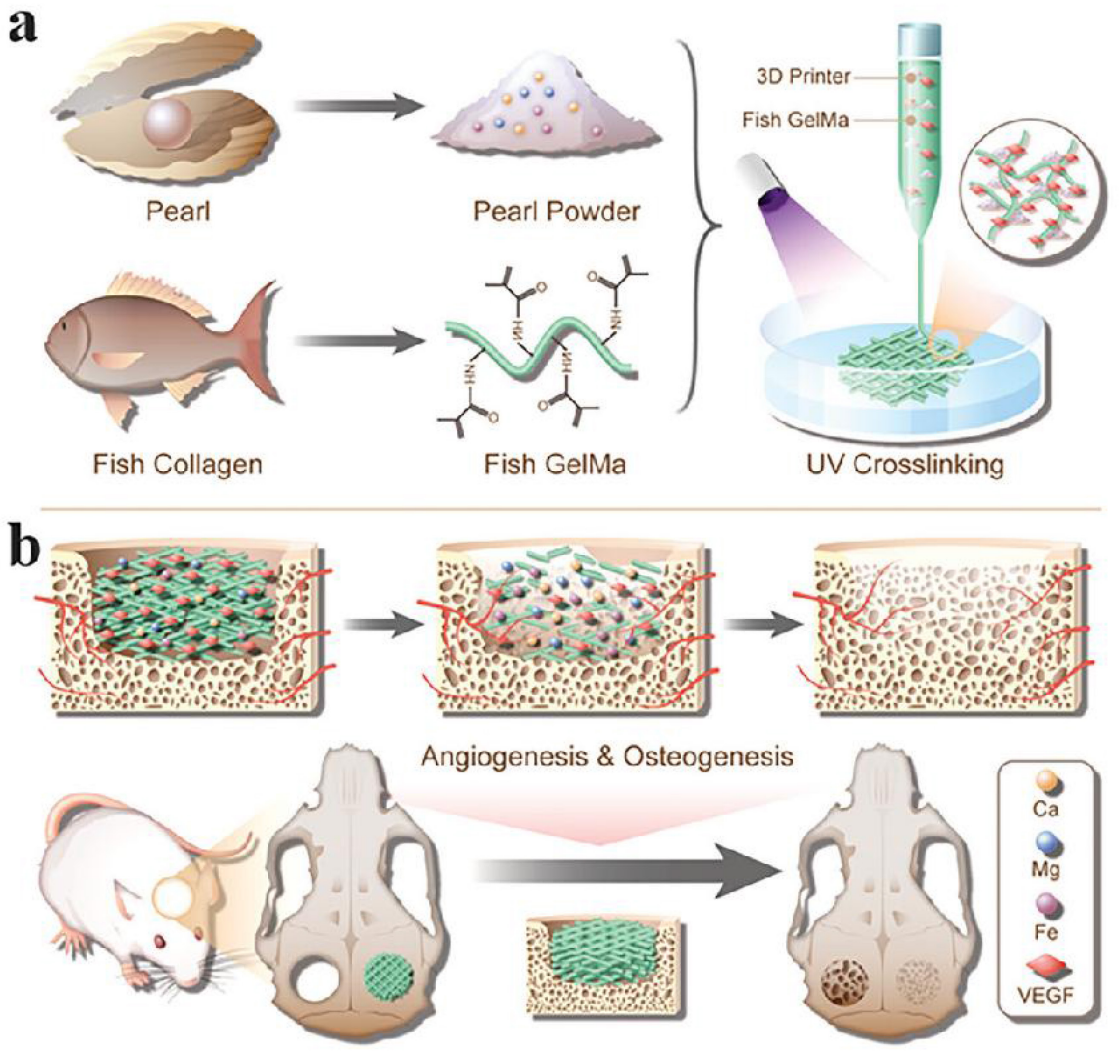
Schematic diagram of PP hybrid bioactive scaffold from microfluidic 3D printing for bone regeneration. (A)The composition and microfluidic 3D printing of PP hybrid bioactive scaffold. (B) The application of PP hybrid bioactive scaffold in bone regeneration. Under Creative Commons Attribution https://creativecommons.org/licenses/by/4.0/ Copyright 2023.

VEGF stands as a potent cytokine that fosters cell growth and angiogenesis by stimulating the proliferation and survival of endothelial cells and enhancing vascular permeability. VEGF is expressed in vascularised tissues and plays a crucial role in both normal and pathological angiogenesis 98. The development of biologically active scaffolds that mimic the ECM of bone tissue is important for bone regeneration. A novel pearl powder (PP) mixed fish GelMA hydrogel scaffold, inspired by bone tissue composites and loaded with VEGF for bone regeneration, has been proposed by researchers 78. By combining microfluidics with 3D printing, the composition and structure of the hybrid scaffold can be precisely adapted for clinical use. The fusion of fish skin GelMA and PP results in a hybrid scaffold with excellent biocompatibility, cell adhesion, and osteogenic differentiation capacity. Controlled release of VEGF facilitates angiogenesis. In a rat cranial defect model, this scaffold expedited bone regeneration through the synergistic effects of osteogenesis and angiogenesis (Figure 3). In another study, a heterogeneous biomimetic structure scaffold was constructed employing a 3D printed mould, simulating the outer/inner periosteum and intermediate bone matrix of natural long bones. Through the modification of mesoporous bioglass nanoparticles (MBGNs), the structural stability and osteogenic capacity of the middle layer of the scaffold were shown to be bolstered. Conversely, the incorporation of GelMA into the VEGF-loaded liposomes facilitated the controlled release of angiogenic factors from the inner and outer layers of the scaffold. This heterogeneous scaffold structure is reported to effectively guide bone regeneration and restoration of the natural bone anatomical structure 103.

**Figure 4:**
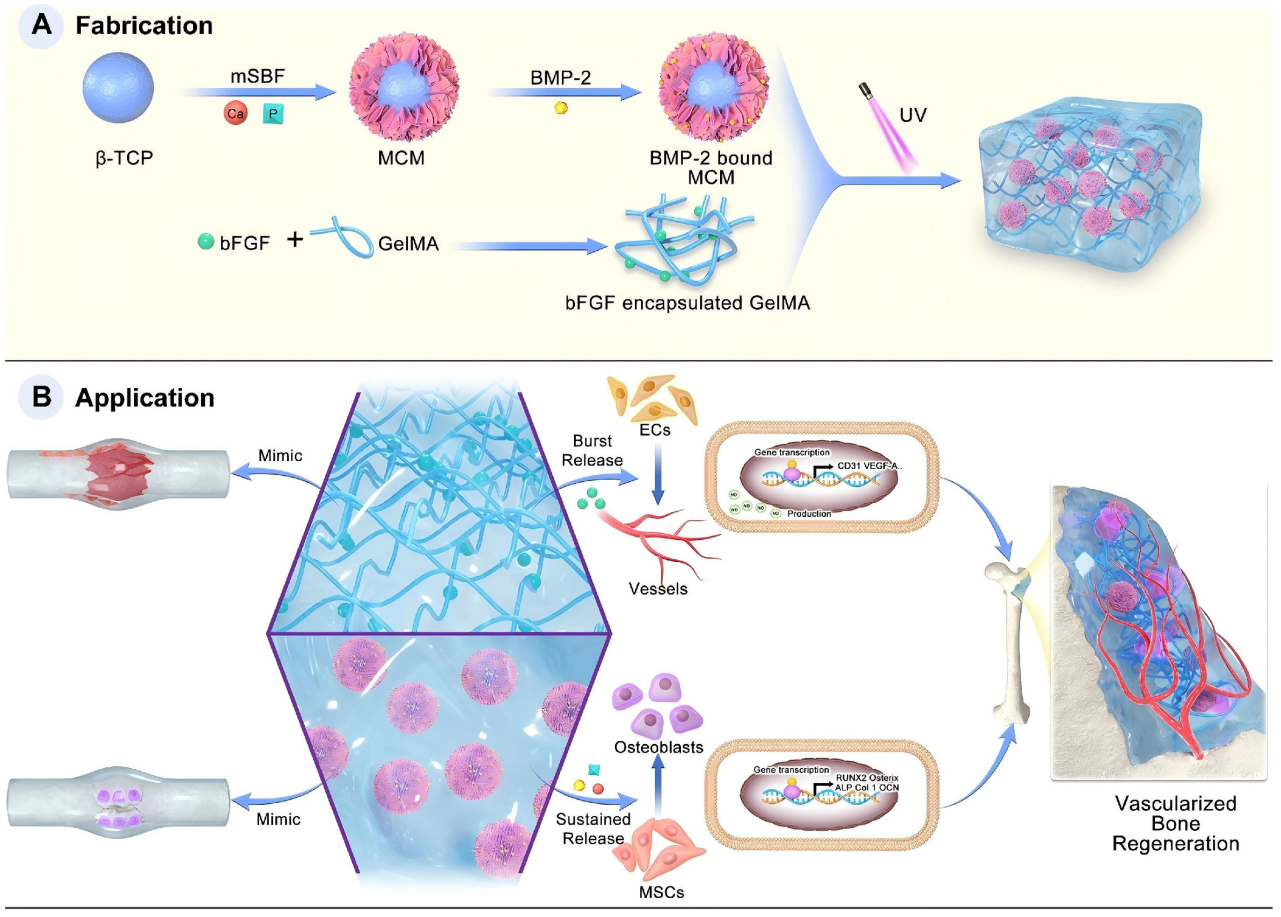
Schematic illustration of the fabrication and application of the F-G/B-M hybrid hydrogel. Under Creative Commons Attribution https://creativecommons.org/licenses/by/4.0/ Copyright 2023.

FGF plays a pivotal role in bone formation and repair, and it is involved not only in embryonic bone development but also in fracture healing and joint cartilage formation 100. To emulate the cascade process of natural bone healing, Zhou *et al.* 104 have concentrated on integrating angiogenesis and osteogenesis using a hybrid dual-factor delivery system to achieve vascularised bone formation. In their study, basic fibroblast growth factor (bFGF) was encapsulated into GelMA to mimic the angiogenic signalling during the inflammatory and soft callus stages of bone healing. BMP-2 binds to mineral-coated microparticles (MCM) to simulate osteogenic signalling during the hard callus and bone remodelling stages. This design achieved the coordinated release of high initial concentrations of bFGF, along with the sustained release of BMP-2 and inorganic ions, fostering a well-coordinated osteogenic and angiogenic effect for bone regeneration. *In vitro* experiments demonstrated that this hybrid hydrogel significantly enhanced the formation of vascular systems by HUVECs and promoted the osteogenic differentiation of BMSCs. This underscores the potential of this system to promote bone repair and regeneration (Figure 4).

### Exosomes

Exosomes, minute vesicles measuring approximately 40-100 nm in diameter, are generated within cells and released into the extracellular milieu through the fusion of multivesicular bodies with the cell membrane 105. These microvesicles are replete with diverse bioactive substances and serve as pivotal carriers for intercellular signalling and interactions, thus playing a central role in tissue repair and regeneration. Exosomes facilitate the transfer of specific proteins, miRNAs, and bioactive factors, thereby promoting the differentiation of mesenchymal stem cells into osteoblasts by activating distinct signalling pathways that upregulate genes associated with osteogenesis, thereby fostering bone defect repair and promoting bone regeneration 105.

**Figure 5:**
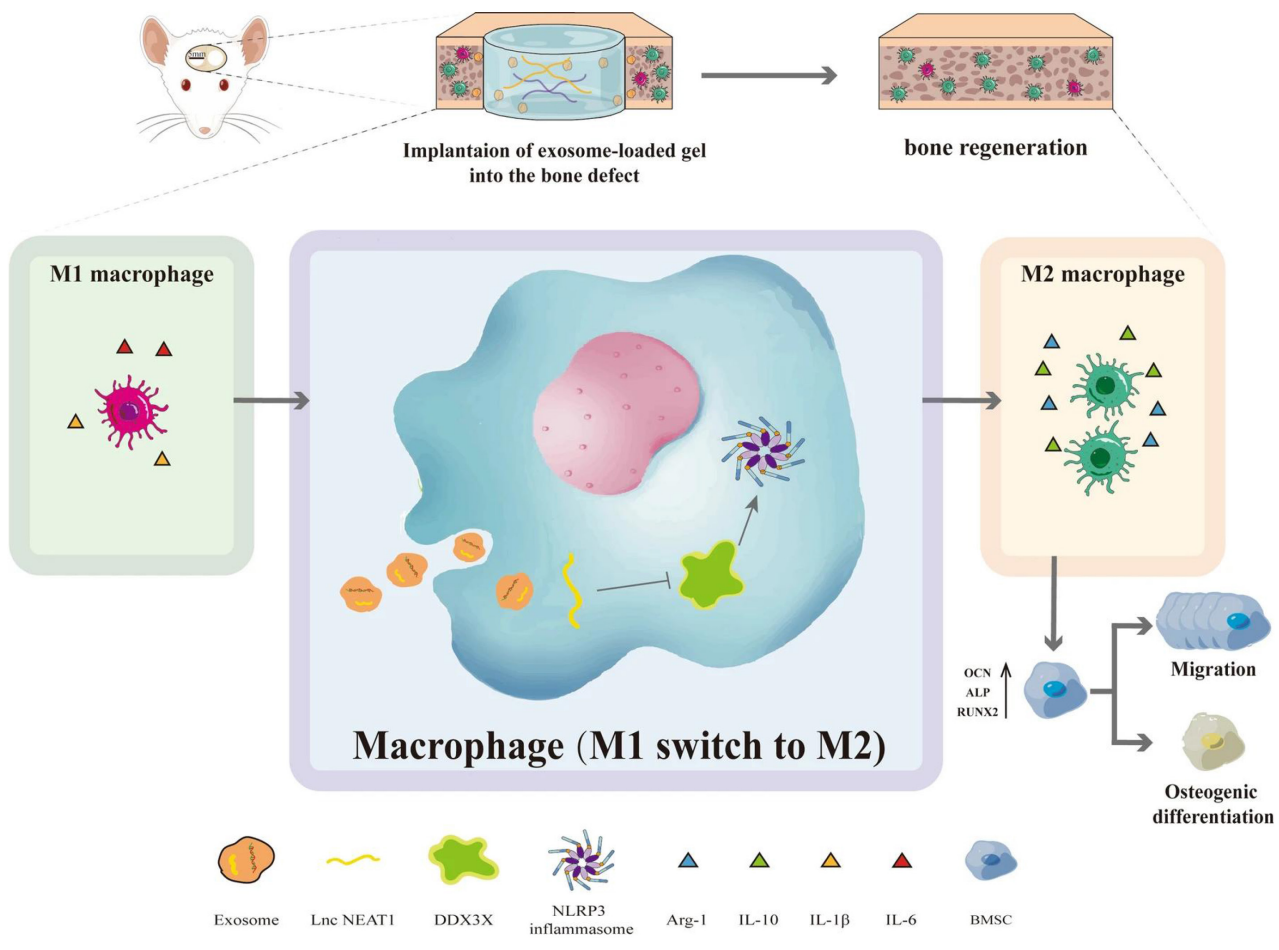
Schematic of the HUVECs derived exosomal NEAT1 mediated bone regeneration mediated by macrophage polarization via DDX3X/NLRP3 axis. Under Creative Commons Attribution https://creativecommons.org/licenses/by/4.0/ Copyright 2023.

Particularly noteworthy is the pretreatment of adipose-derived stem cell (ADSC) exosomes under hypoxic conditions, which has garnered attention because of the heightened secretion and functionality of exosomes. Li *et al.* 82 indicated that hypo-ADSC-Exos harbour a crucial miRNA, miR-21-5p, which is a major regulator of angiogenesis. Hypo-ADSC-Exos have been demonstrated to promote proliferation, migration, and angiogenesis in HUVECs *in vitro*. They reported that inhibition of miR-21-5p effectively attenuates proangiogenic effects mediated by hypo-ADSC-Exos. Mechanistically, their study demonstrated that exosomes from hypo-ADSCs target SPRY1 within HUVECs to exert their regulatory effects, thereby promoting activation of the PI3K/AKT signalling pathway. Notably, silencing SPRY1 enhances PI3K/AKT activation in HUVECs and promotes proliferation, migration, and angiogenesis. The culmination of the above study involved an *in vivo* demonstration, validating that loading hypo-ADSC-Exos into GelMA significantly augments local H-type angiogenesis and concomitant bone regeneration, attributed to the modulation of SPRY1 by exosomes. Additionally, HUVEC-derived exosomes carrying NEAT1 substantially enhanced M2 polarisation and alleviated LPS-induced inflammation *in vitro*. Exosome-induced macrophage-conditioned medium indirectly promotes the migration and osteogenic differentiation of BMSCs. Mechanistically, exosomes carrying NEAT1 significantly downregulate the expression of DDX3X and NLRP3 in HUVECs. *In vivo*, HUVEC-derived exosomes markedly increase pro-inflammatory cytokines (IL-6 and IL-1β) and anti-inflammatory cytokines (IL-10), thus ameliorating LPS-induced inflammation. Subsequently, encapsulating HUVEC-derived exosomes in alginate/GelMA interpenetrating polymer network (IPN) hydrogels promotes bone regeneration and angiogenesis, augments M2 polarized macrophage infiltration, and reduces NLRP3 expression, as reported in a rat cranial defect model 84 (Figure 5).

## Antibacterial strategy

In clinical practice, addressing infectious bone defects and fostering bone regeneration poses a formidable challenge. Upon bacterial infiltration into the bone tissue, the ensuing production of acidic metabolites and the provocation of a robust immune response dampens osteoblastic activity and impedes the restitution of bone defects 106. Furthermore, bacterial pathogens release toxins, virulent factors, and cytotoxic substances that deleteriously impact the bone matrix, precipitating nerve and blood vessel necrosis and severely hindering bone regeneration. Consequently, interest in antimicrobial hydrogels for bone tissue engineering has rapidly increased. By employing various antimicrobial agents such as antibiotics, metal nanoparticles, and botanically active compounds, these hydrogels, specifically designed for bone restoration, can meticulously and efficiently eradicate bacteria, thereby effectively preventing infections and reducing postoperative complications 107,108. This review systematically delineates the antimicrobial strategies anticipated for GelMA hydrogels, as outlined in Table 3.

### Antibiotics

The conveyance of antibiotic molecules through carriers represents a straightforward and efficacious traditional dosing approach, affording localised administration to circumvent the systemic drug tolerance hazards inherent to intravenous administration 140. Currently, antibiotics are broadly classified into seven categories: β-lactam antibiotics (encompassing penicillins and cephalosporins), macrolide antibiotics (such as erythromycin and azithromycin), aminoglycoside antibiotics (including gentamicin and etimicin), tetracycline antibiotics (such as tetracycline), lincosamide antibiotics (such as clindamycin), chloramphenicol antibiotics (such as chloramphenicol), and peptide antibiotics (such as vancomycin).

β-Lactam Antibiotics: The mode of action of β-lactam antibiotics primarily involves targeting bacterial penicillin-binding proteins (PBPs), thereby inhibiting the enzyme responsible for peptidoglycan synthesis in the bacterial cell wall. This inhibition impairs peptidoglycan synthesis, resulting in the formation of defective bacterial cell walls. Consequently, external water penetrates the cells, inducing swelling and rupture, which triggers bacterial autolysin activity and ultimately results in bacterial death. Vigata *et al.* 109 devised a hydrogel drug delivery system (termed GelMA-DDS) based on GelMA for the localised delivery of the broad-spectrum antibiotic cefazolin, with the aim of preventing and treating postoperative infections in surgical wounds. The researchers fabricated GelMA hydrogels with polymer concentrations ranging from 5% to 15% w/v and loaded them with doses of cefazolin ranging from 3μg to 90μg, followed by facile photocrosslinking for solidification. The findings demonstrated that all the GelMA groups exhibited a remarkable drug encapsulation efficiency of 99 %. Additionally, cefazolin dispensed by GelMA showed dose-dependent antimicrobial efficacy against *S. aureus* in broth and diffusion assays. The loaded cefazolin GelMA-DDS provides a highly adaptable and user-friendly localised delivery system and a novel modality for preventing and treating postoperative infections in surgical wounds.

Macrolide Antibiotics: The macrolide antibiotics bind to the 50S subunit of bacterial ribosomes, which are responsible for protein synthesis. Upon binding, they impede the translocation of bacterial proteins, thereby disrupting the bacterial protein synthesis process. Ayoub *et al.* 110 devised a novel photocrosslinkable azithromycin (AZ)-loaded GelMA fibre using electrospinning technology, which served as a localised and biodegradable drug delivery system for managing dental pulp infections. Fibres were fabricated at three different concentrations: GelMA+5%AZ, GelMA+10%AZ, and GelMA+15%AZ. Fibres comprising GelMA and GelMA+10%AZ exhibited the highest average diameters. The incorporation of AZ reduced the tensile strength of the GelMA-based fibres. Furthermore, the GelMA+15%AZ fibres demonstrated the most pronounced inhibition of bacterial growth. Importantly, the presence of AZ at all tested concentrations did not elicit a significant toxic response in their study. Additionally, findings from the subcutaneous rat model revealed abundant vascular formation in the experimental group, along with attenuated inflammation and mature collagen fibres intertwined with the engineered fibres, signifying promising prospects for tissue engineering.

Peptide Antibiotics: Chemically synthesised antibiotic peptides typically employ a mechanism of action similar to that of natural AMPs, primarily targeting the bacterial cell membrane to exert bactericidal effects. This membrane-damaging mechanism disrupts the integrity of the bacterial cell membrane, leading to pore formation and the subsequent leakage of cellular contents. Qian *et al.* 111 encapsulated vancomycin (Van) in poly(lactic-co-glycolic acid) (PLGA) microspheres using liquid encapsulation technology, which were then loaded into GelMA hydrogels. These microsphere-loaded hydrogels were embedded into additively manufactured porous tantalum (AM-Ta) structures to create a composite scaffold—Ta/GelMA hydrogel/PLGA/Van—for treating infectious bone defects. The physicochemical characterisation of this scaffold revealed a vancomycin-release cycle lasting for over two weeks. Subsequent biological experiments confirmed the excellent biocompatibility and antibacterial and osteoconductive properties of the scaffold, highlighting its considerable potential for clinical applications.

Tetracycline Antibiotics: Tetracycline antibiotics exert their antimicrobial effects by forming reversible complexes with the 30S subunit of bacterial ribosomes, thereby inhibiting protein synthesis. Zhang *et al.* 112 developed a modular microneedle (MN) patch for the effective delivery of antibiotics and cytokines to the local gingival tissue to achieve immune regulation and tissue regeneration. The MN patch comprises two components: a rapidly dissolving gelatine membrane releasing tetracycline and a biodegradable GelMA MN containing tetracycline-loaded poly(lactic-co-glycolic acid) nanoparticles and cytokine-loaded silica microparticles for sustained release. Experimental results demonstrated that antibiotic release could completely inhibit bacterial growth, while the release of TGF-β and IL-4 induced anti-inflammatory macrophage reprogramming and promoted regulatory T cell formation *in vitro*. When applied to the periodontal tissue *in vivo*, the MN patch suppressed proinflammatory factor production, promoted proregenerative signals, and facilitated tissue healing, highlighting its potential for local immune regulation in tissue regenerative therapy.

Lincosamide Antibiotics: Lincomycin, a lincosamide antibiotic produced by Lincomyces, acts on sensitive bacterial ribosomes by binding to the central loop of the 50S subunit 23SrRNA gene, preventing elongation of the peptide chain and inhibiting bacterial cell protein synthesis. Ribeiro *et al.* 113 used electrospinning to incorporate clindamycin (CLIN) and metronidazole (MET) into polymer solutions to prepare fibre mats. The mats were then processed by low-temperature grinding to obtain fibre particles containing CLIN or MET. These particles were used to modify the GelMA hydrogel. Morphological characterisation of the electrospun fibres and particles obtained by low-temperature grinding was performed using scanning electron microscopy (SEM). Furthermore, the swelling, degradation, and toxicity of the experimental hydrogels toward dental pulp stem cells were tested, and their antibacterial efficacy was assessed using agar diffusion and biofilm inhibition tests. The results showed that GelMA hydrogels modified with fibre particles containing antibiotics showed increased swelling and degradation rates. Dental pulp stem cell viability slightly decreased but did not show significant toxicity (cell viability >50%). All hydrogels containing antibiotic-loaded fibre particles exhibited antibiofilm activity with almost complete elimination of viable bacteria in the dentin matrix.

### Antimicrobial peptides

AMPs serve as critical immune defence molecules in multicellular organisms, showcase broad-spectrum bactericidal effects, and are emerging as promising candidates for novel antimicrobial therapies. Currently, over 30 AMP drugs are undergoing clinical studies worldwide, highlighting their potential clinical applications. Recent studies have revealed synergistic effects between certain AMPs and conventional antibiotics. By combining AMPs with antibiotics, it is possible to reduce antibiotic dosage, minimise side effects, mitigate the emergence of antibiotic-resistant strains, and significantly enhance the overall therapeutic efficacy of antibiotics 141,142. This synergy represents a promising avenue for combating bacterial infections and addressing the challenges posed by antibiotic resistance.

**Figure 6:**
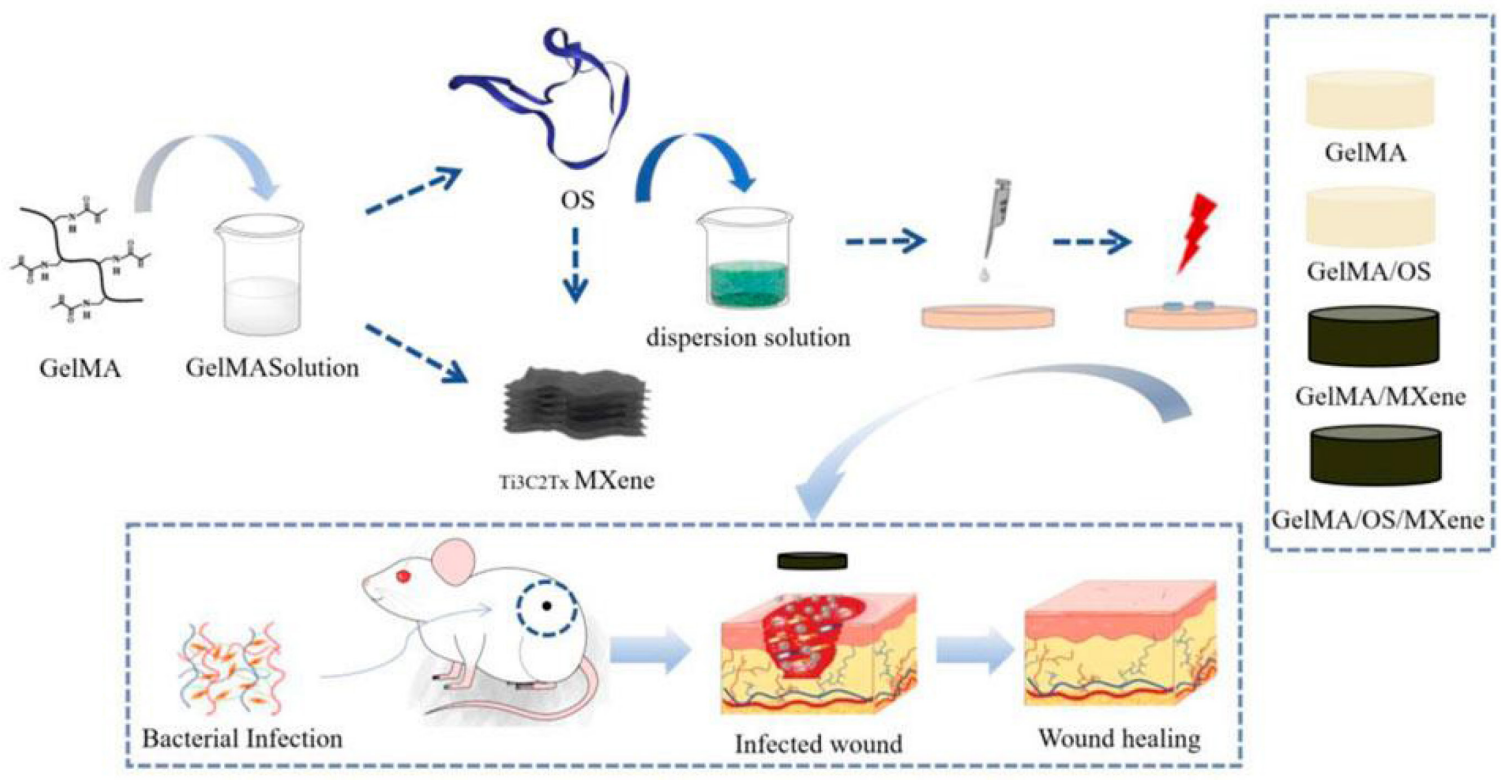
Schematic diagram of the experimental procedure. This experiment involves preparing GelMA solution and TBCZ@MXene dispersion solution, as well as GelMA/OS and GelMA/MXene composite materials. Subsequently, a bacterial infection wound model on mice is established, followed by the application of these materials to the infected wounds. Finally, the antibacterial effects of these materials and their impact on wound healing are assessed aiming to explore the effectiveness of GelMA-based composite materials in treating bacterial infection wounds. Under Creative Commons Attribution https://creativecommons.org/licenses/by/4.0/ Copyright 2023.

Ren *et al.* 115 developed a multifunctional conductive hydrogel dressing using GelMA, Ti3C2, and collagen-bound AMPs (V-Os). Electrical stimulation (ES) promotes skin wound healing. However, traditional ES strategies based on single electrodes may struggle to achieve uniform coverage over the entire wound area, affecting treatment outcomes because of mechanical property mismatches between the electrodes and the wound tissue. This dressing aimed to improve wound management by incorporating GelMA, Ti3C2 for conductivity, and modified AMPs (V-Os) to replace traditional antibiotics. Their study demonstrated that the GelMA@Ti3C2/V-Os hydrogel dressing exhibited excellent conductivity and biocompatibility with sustained bactericidal effects. V-Os were tightly bound to GelMA, thereby prolonging its antimicrobial activity. Cell experiments revealed enhanced fibroblast migration, proliferation, and tissue repair gene expression with dressings, particularly under electrical stimulation. *In vitro*, it promotes wound re-epithelialisation, angiogenesis, immune response mediation, and infection prevention. This dressing shows promise for wound healing because it provides moisture retention, biocompatibility, mechanical strength, and antimicrobial properties. In another study by Liang *et al.* 116, tick-derived AMPs (Os) were encapsulated in a GelMA hydrogel containing MXene nanoparticles. The composite hydrogel exhibited excellent mechanical strength, swelling, degradation, and antimicrobial activity and promoted cell growth, adhesion, and antimicrobial peptide activity expression of AMPs. In full-thickness rat wound-healing experiments, the composite hydrogel accelerated wound closure, reduced inflammation, and promoted epithelial cell formation and maturation. Incorporating AMPs into GelMA hydrogels is a feasible strategy for developing high-quality antimicrobial wound dressings with strong tissue regenerative capacity (Figure 6).

More recently, poly-L-lysine, an AMP, has gained attention owing to its high safety profile and broad-spectrum inhibitory effects against Gram-positive and Gram-negative bacteria, fungi, and other microbes. Its thermal stability and good water solubility make it suitable for a wide range of applications in the food preservation and medical fields, particularly as a coating material. Chen *et al.* 117 explored the advantages of polyether ether ketone (PEEK) in self-initiated graft polymerisation and the application of hydrogels in bone tissue engineering. They constructed a hydrogel coating (GPL) on the surface of PEEK using UV-induced crosslinking, composed of GelMA, methacryloyl-modified ε-poly-l-lysine (ε-PLMA), and laponite. This coating not only enhanced the hydrophilicity of PEEK but also exhibited slow-degrading characteristics, retaining approximately 80% of the coating after eight weeks of incubation in PBS. *In vitro* studies showed that PEEK-GPLs enhanced the viability and adhesion of human umbilical cord Wharton jelly mesenchymal stem cells (hWJ-MSCs) compared to plain PEEK. The micrometre-scale three-dimensional surface structure of the GPL coating and the synergistic effect of laponite significantly improved the ability of PEEK-GPL to induce hWJ-MSC osteogenic differentiation with increased alkaline phosphatase activity, matrix mineralisation, and osteogenic gene expression. Furthermore, PEEK-GPL demonstrated antimicrobial activity against *S. aureus* and *E. coli*, which lasted until the hydrogel fully degraded due to the covalent crosslinking of ε-PLMA in the coating. Infection resistance and bone integration are critical considerations for antimicrobial titanium implants. To address these issues, Zhang *et al.* 118 prepared a titanium surface with a micro-nano structure using dual-acid treatment and anodic oxidation and then coated it with a GelMA hydrogel loaded with GL13K. *In vitro* cell experiments, including observations of the cell cytoskeleton, cell viability, alkaline phosphatase activity, mineralisation, and osteogenic gene expression, demonstrated the biocompatibility of the released hydrogel-modified micro/nano-titanium peptide, promoting osteoblast differentiation. *In vitro* antimicrobial experiments showed that it could also inhibit the growth of gram-positive bacteria (*S. aureus*) and gram-negative bacteria (*E. coli*) by releasing GL13K. These studies validated the modification of titanium surfaces to develop antimicrobial titanium implants, providing a new solution for bone tissue engineering.

### Plant-derived antimicrobial agents

Plant-derived antimicrobials are bioactive compounds procured from botanical sources via diverse extraction techniques, including physical, chemical, and biological methods. These compounds are broadly classified as polyphenols, anthraquinones, terpenoids, and alkaloids, each harbouring chemical constituents such as phenolic, ether, terpenoid, and ketone groups. Recently, the efficacy of plant extracts against external pathogens has emerged as a focal point of research. Owing to their eco-friendliness, minimal environmental impact, and limited adverse effects, plant-derived antimicrobials have been widely utilised in various sectors such as cosmetics, natural preservatives, and animal feed production 143,144. The antimicrobial modalities exhibited by these compounds are as follows: 1. Direct disruption of bacterial cell integrity, leading to enhanced membrane permeability and subsequent leakage of intracellular components; 2. Genetic impairment of bacterial DNA, 3. Perturbation of bacterial intracellular enzymatic cascades, culminating in enzyme denaturation and functional incapacitation; and 4. Alterations in bacterial oxidative respiratory pathways 143.

### Polyphenols

Polyphenols exhibit notable efficacy in impeding the proliferation of both bacteria and fungi, and their antimicrobial potency is positively correlated with their concentration. Tea polyphenols, primarily catechins, are widely used as botanical polyphenols 143. These lipophilic compounds can permeate the bacterial cells and exert antimicrobial effects. Dong *et al.* 119 engineered a dual-network hydrogel through the synergy of hydrogen bonding interactions between tea polyphenols (TP) and glycerine and photocrosslinked N-acryloylaminoacetamide (NAGA), GelMA, and nanoclay laponite (NGL) hydrogels. The introduction of glycerine hindered the diffusion of TP into the NAGA/GelMA/laponite (NGL) hydrogel, thus preventing excessive crosslinking and facilitating the formation of a homogeneous network. This hydrogel demonstrated exceptional moisture retention, sustaining 84% moisture over 28 days. Moreover, owing to the hygroscopic nature of glycerine, the mechanical robustness (0.73-1.14 MPa) and tensile strain (207%-353%) of the hydrogel were further augmented after 14 days in an open environment. The hydrogel exhibited remarkable UV protection and antioxidant capabilities, effectively mitigating oxidative stress at the wound sites and expediting wound healing. Additionally, antimicrobial efficacy against *E. coli* and *S. aureus* was evident in the hydrogel wound dressings. Due to its dual-network design, the NGLG20/TG hydrogel dressing accelerated wound healing by promoting wound closure, angiogenesis, and collagen deposition, offering a unique wound management approach.

**Figure 7:**
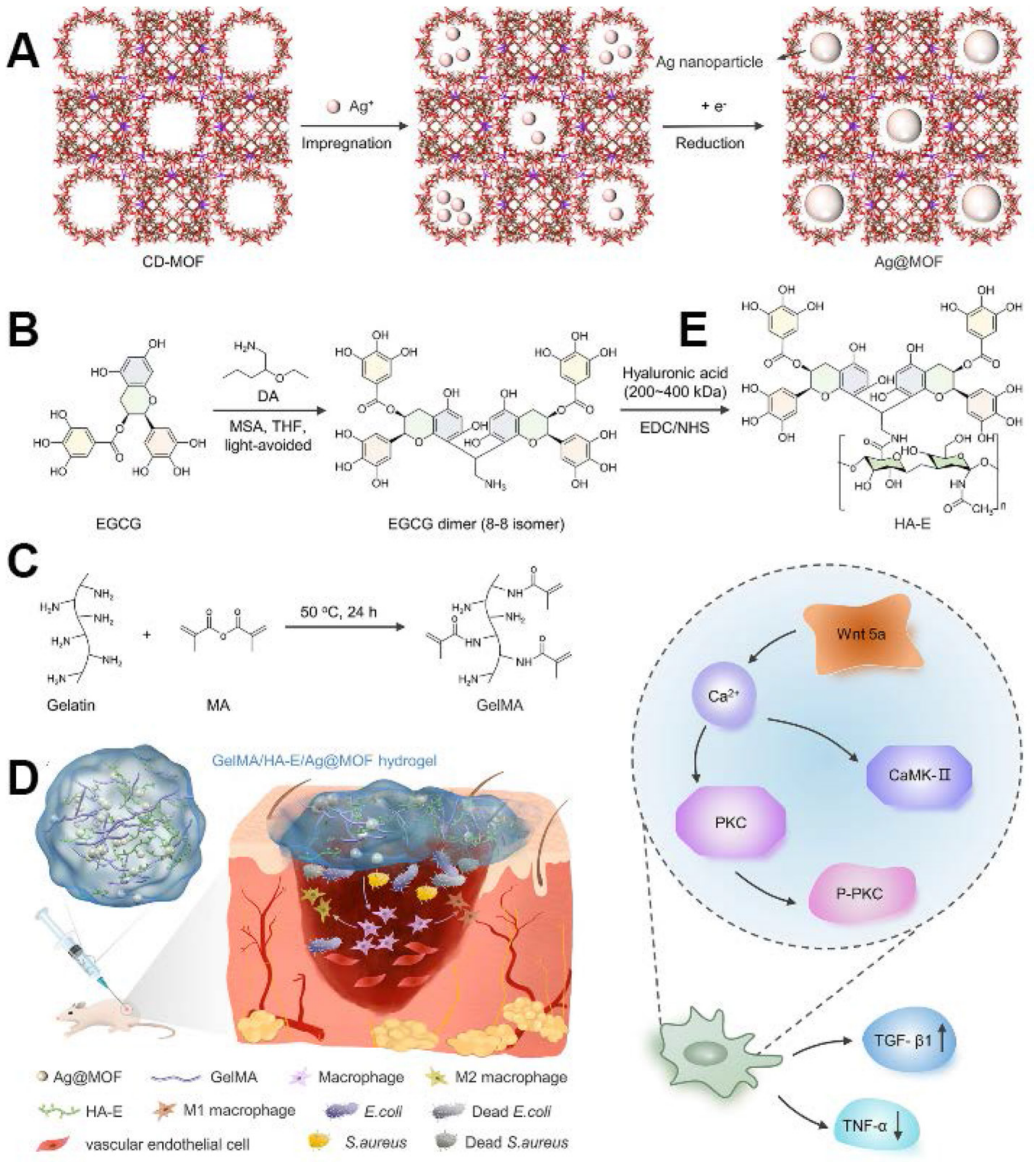
(A) Schematic of CD-MOF template guided synthesis of Ag@MOF. (B) Schematic diagram of synthesis of HA-E. (C) Schematic ofsynthesis of GelMA. (D) Schematic of infectious burn wound healing process including bacterium invasion, macrophages polarization and pro-inflammation cytokines release. (E) Schematic diagram of activated noncanonical Wnt pathway in GelMA/HA-E/Ag@MOF hydrogel group. Copyright 2022, with permission from Wiley.

Epigallocatechin gallate (EGCG), one of the most potent constituents of tea polyphenols, has garnered significant attention for its anticancer, antimutagenic, and cardiovascular protective properties, as well as its capacity to modulate the endocrine and immune systems. Xiong *et al.* 120 innovated a novel GelMA hydrogel system incorporating silver nanoparticles encapsulated in γ-cyclodextrin metal-organic frameworks (Ag@MOF) and hyaluronic acid-epigallocatechin gallate (HA-EGCG). The GelMA/HA-EGCG/Ag@MOF hydrogel exhibited favourable physical attributes and sustained Ag + release. Furthermore, the hydrogel exhibited excellent biocompatibility and facilitated the polarisation of macrophages from M1 to M2 phenotype. *In vivo* evaluations of wound healing showed the hydrogel's ability to curtail bacterial proliferation, expedite wound closure, stimulate early angiogenesis, and modulate immune responses. Subsequent findings highlighted the significant activation of the non-canonical Wnt signalling pathway in the GelMA/HA-EGCG/Ag@MOF hydrogel-treated group (Figure 7).

#### Terpenoids

Terpenoids, which are prevalent constituents of plant essential oils, resins, and pigments, exhibit diverse biological functions, including anti-inflammatory, antitumour, anti-HIV, and lipid-lowering effects 143. The principal sources of terpenoids are *Artemisia annua* and *Prunus persicae*. Moghtaderi *et al.* 122 have harnessed thymol, a terpenoid-derived antimicrobial agent, to treat antibiotic-resistant microbes. To facilitate efficient thymol delivery, a hydrophilic polymer hydrogel with exceptional biocompatibility was synergistically amalgamated using niobium-based technology for thymol encapsulation, culminating in the formation of a GelMa composite system. Thereafter, optimisation efforts focused on enhancing the thymol capture efficiency, minimising the particle size, and reducing the polydispersity index, resulting in Nio-Thymol@GelMa achieving a peak thymol release of 60% and 42% in media with pH values of 6.5 and 7.4, respectively. Moreover, Nio-Thymol@GelMa demonstrated superior antimicrobial and anti-biofilm efficacy against both Gram-negative and Gram-positive bacteria compared with Nio-thymol and free thymol. Notably, Nio-Thymol@GelMa significantly augmented the migration of human dermal fibroblasts *in vitro* and upregulated the expression of pivotal growth factors (e.g., FGF-1) and MMPs (e.g., MMP-2 and MMP-13). Consequently, Nio-Thymol@GelMa has emerged as a promising novel thymol drug formulation, offering considerable potential for enhancing wound-healing processes and augmenting antimicrobial efficacy.

**Figure 8:**
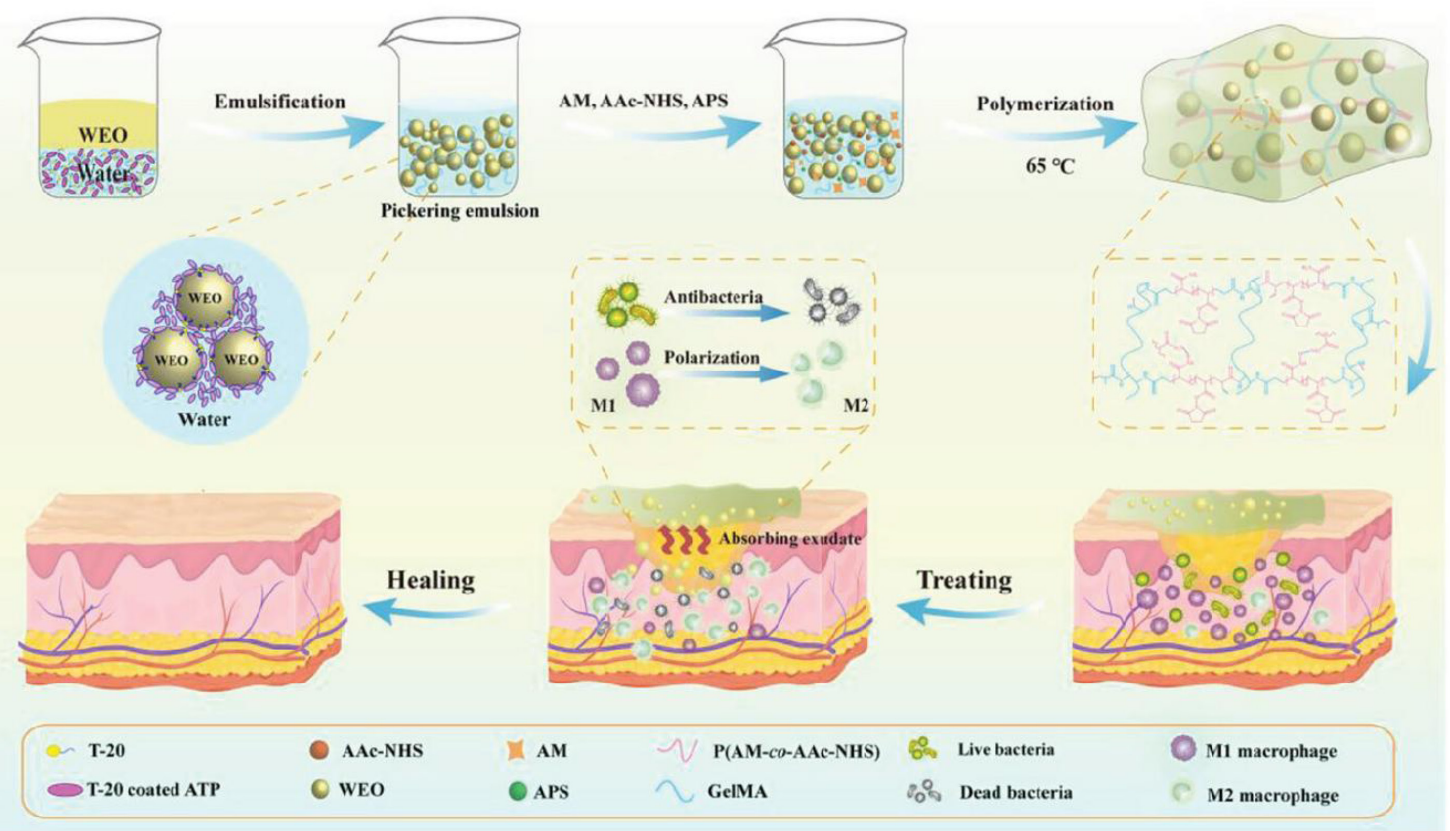
The preparation process diagram of HD-WEO and the mechanism to promote healing of infected diabetic wound. Reproduced from reference 124 Copyright 2023, with permission from Wiley.

In response to the escalating conundrum of multidrug-resistant bacteria, Wang *et al.* 123 harnessed *Artemisia annua* essential oil (WEO) encapsulated within a GelMA, acrylamide (AM), and acryloyl N-hydroxysuccinimide (AAc-NHS) polymerisation process using the O/W-Pickering emulsion technique to form a multifunctional hydrogel dressing (HD-WEO). Compared to conventional emulsions, Pickering emulsions not only bolstered the encapsulation stability of the WEO but also fortified the tensile and swelling properties of the hydrogel. The diverse bioactive constituents of the WEO exhibit broad-spectrum antimicrobial activity against *S. aureus*, *E. coli*, and methicillin-resistant *S. aureus* (MRSA). Furthermore, HD-WEO facilitated the polarisation of macrophages from the M1 to the M2 phenotype. HD-WEO effectively promoted collagen deposition and neoangiogenesis, thereby expediting the healing of MRSA-infected diabetic wounds (Figure 8).

#### Anthraquinone

Anthraquinone, a naturally occurring quinone compound, comprises various reduced products and dimers, including anthrahydroquinone, oxidised anthrahydroquinone, and anthraquinone, along with their glycosides. Within the natural realm, anthraquinones are prevalent in the metabolic byproducts of higher plants, lower plants, lichens, and fungi, showing diverse functionalities such as haemostasis, antimicrobial activity, purgative effects, and diuretic properties. Namazi *et al.* 124 fabricated a highly biocompatible biomaterial capable of eradicating dental pulp infections and modulating inflammation by integrating aloe vera, which is primarily enriched with aloe emodin, an antimicrobial constituent, into photo-crosslinkable GelMA nanofibers. These efforts yielded stable GelMA/AV nanofibers with an optimal (70:30) ratio achieved via electrospinning. These GelMA/AV (70:30) nanofibers showed remarkable antimicrobial efficacy against *Enterococcus faecalis*, maintaining sustained activity for over 14 days, along with notable biofilm reduction and minimal cytotoxicity. Furthermore, these nanofibers exhibited pro-healing, anti-inflammatory, and immunomodulatory properties.

### Metal nanoparticles

Antimicrobial agents can be categorised into three main types: inorganic, organic, and natural. Organic antimicrobials are often disregarded because of their limited heat resistance, elevated toxicity, and propensity to foster antibiotic resistance. Similarly, natural antimicrobials, which are limited by their source, lack widespread applicability. Inorganic antimicrobials, predominantly metal-based, are favoured because of their expansive antimicrobial spectrum, exceptional antibacterial efficacy, and the absence of resistance development. Metallic ions have potent inhibitory and bactericidal effects, with certain metal ions posing no harm to human health 145. Typically, the antimicrobial activity of metal ions follows this order: Ag^+^ > Co^2+^ ≥ Ni^2+^ ≥ Al^3+^ ≥ Zn^2+^ ≥ Cu^3+^ = Fe^3+^ > Mn^2+^ ≥ Sn^2+^ ≥ Ba^2+^ ≥ Mg^2+^ ≥ Ca^2+^ 146. The mechanisms of antimicrobial action can be broadly classified as ionic, degradative, oxidative, or substitutional.

#### Ionic antimicrobials

Ionic antimicrobials represent a widely explored and utilised category of antimicrobial agents distinguished by their capacity to interact with bacteria through diverse mechanisms such as electrostatic attraction and ionic dissolution 147. Metals such as copper and silver exemplify prominent instances of ionic antimicrobials, which are renowned for their broad-spectrum antimicrobial activity and efficacy. Xiang *et al.* 125 conducted a study on the augmentation of the antimicrobial properties of injectable wound dressings using multifunctional ionic silver nanoparticles (AgNPs). This investigation entailed the development of a biomimetic polymer copolymerised with sulfobetaine and catechol groups (PSBDA), which served as a stabilising strategy to streamline the synthesis of AgNPs. The phenolic constituent of PSBDA demonstrated the ability to reduce AgNO3 in alkaline solutions and immobilise PSBDA on the surface of AgNPs. This yielded AgNPs characterised by a uniform size distribution and markedly enhanced stability, which is pivotal for sustaining antimicrobial efficacy in physiological milieus. The precursor hydrogel exhibited excellent injectability and promptly responded to UV-induced *in situ* gelation. Compared to hydrogels without amphoteric ionic AgNP modification, the AgNP-incorporated hydrogel exhibited heightened antimicrobial effectiveness in both *in vitro* and *in vivo* assays. Furthermore, amphoteric ionic modifications improved hemocompatibility and biocompatibility. This hydrogel dressing facilitated the resolution of inflammation and rapid re-epithelialisation, thereby expediting healing in a full-thickness rat wound model.

#### Degradative antimicrobials

As research on degradative antimicrobial mechanisms has progressed, pure Mg and Mg alloys have increasingly come under the scrutiny of researchers. Robinson *et al.* 148 have confirmed the bactericidal effect of Mg, suggesting that its antimicrobial environment is alkaline. Additionally, the bactericidal properties of Mg-based metals have been investigated in animal experiments. These studies revealed that the bactericidal action of Mg-based metals differs from that of copper, silver, and zinc, as Mg-based metals do not rely on ionic effects. Instead, Mg-based metals exert their antibacterial effects through self-degradation in the body or environment, significantly increasing the environmental pH, thereby altering the conditions for bacterial growth and ultimately leading to bacterial eradication 149. Researchers have also utilised polydopamine (PDA), which interacts with the amino groups in polyacrylamide (PAM) through its amino and catechol groups, to form a network with PAM. Magnesium ions (Mg^2+^) were incorporated into hydrogels to promote cell proliferation, differentiation, and tissue regeneration. The mixed crosslinking of covalent bonds and reversible non-covalent bonds in the PDA-PAM/Mg^2+^ composite endows it with excellent self-healing and adhesive properties. Under near-infrared radiation, PDA in the hydrogel demonstrated remarkable photothermal efficiency and antibacterial activity. The introduction of Mg^2+^ enhances the photostability and recyclability of the photothermal effect 150.

However, research on the degradation mechanisms of antimicrobial metals remains limited. This scarcity may be attributed to the rapid degradation rate of Mg^2+^, which could lead to bacterial infections and adversely affect biocompatibility, thus hindering its clinical development.

#### Oxidative antimicrobials

**Figure 9:**
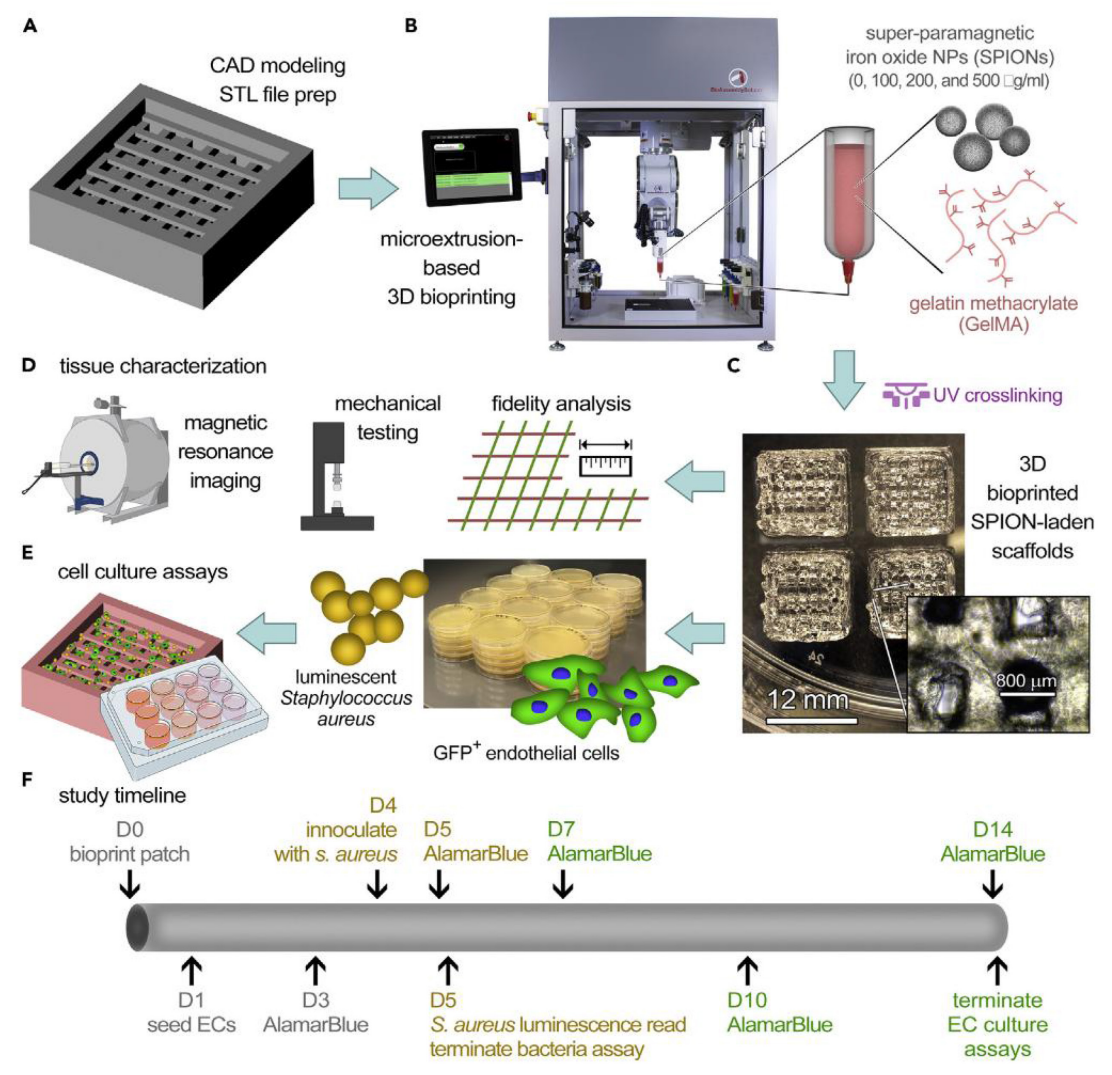
Schematic overview of the experimental design for the fabrication and *in vitro* analysis of bacteriostatic.3D scaffold systems (A-E) A CAD model of the 3D geometry of interest was designed (A) and bioprinted (B) using various GelMA bioinks containing 0 (control), 100, 200, and 500 mg/mL of superparamagnetic iron oxide nanoparticles (SPIONs) to create the 3D Scaffold (C). Bioprinted scaffolds were assessed for printing fidelity and mechanical and MR properties (D), and they were seeded with human cells (fluorescently tagged endothelial cells) and/or bacteria (luminescent *S. aureus*) (E) for *in vitro* 3D culture assays. (F) Timeline for *in vitro* culture assays. Constructs were cultured for 5 days (bacterial and human cell coculture) or 14 days (endothelial cell culture) and examined using AlamarBlue and imaging techniques. In panel (F), grey represents the experimental steps for both bacterial and cell culture assays, brown represents the steps for the bacterial assays; and green represents steps for endothelial cell culture reproduced from reference 128 Copyright 2023, with permission from Elsevier.

Oxidative antimicrobials include metals that may not inherently possess robust antibacterial properties or exhibit limited effectiveness. However, metal oxides can also exhibit potent antimicrobial activity under specific circumstances. Illustrative examples include zinc oxide and iron oxide, which undergo chemical reactions upon exposure to light under specific environmental conditions, culminating in bactericidal effects. Haghniaz *et al.* 126 devised an injectable, photocrosslinkable, and stretchable hydrogel sealant on GelMA fortified with antibacterial ferrimagnetic zinc oxide (ZF) nanoparticles and haemostatic silicate nanoplatelets (SN) for prompt clotting. The hydrogel demonstrated the capacity to diminish the viability of *S. aureus* by over 90% *in vitro*. Incorporating SN (2% w/v) and ZF nanoparticles (1.5 mg/mL) into GelMA (20% w/v) notably increased the bursting pressure of perforated porcine lungs by > 40%. Relative to the commercial haemostatic sealant Evicel, this fortified formulation enhanced tissue-sealing efficacy by approximately 250%. Additionally, in a rat bleeding model, the hydrogel curtailed bleeding by approximately 50%, serving as a biocompatible wound-closure adhesive with dual haemostatic and antibacterial attributes. Another study introduced a novel generation of antimicrobial gelatine bioinks based on GelMA containing varying doses of antimicrobial superparamagnetic iron oxide nanoparticles (SPION). The SPION-loaded GelMA scaffolds showed notable resistance to the proliferation of *S. aureus*. *In vitro* simulations of bacterial contamination on 3D GelMA scaffolds underscored the pivotal role of functionalized scaffolds in impeding bacterial proliferation while preserving cell viability and proliferation 127 (Figure 9).

#### Substitutional antimicrobials

Substitutional antimicrobials operate on the premise of the structural resemblance between metal ions and essential bacterial nutrients. This structural similarity causes confusion, with antibacterial elements substituting for vital nutrients and infiltrating bacterial cells. Consequently, these elements disrupt proteins and enzymes, thereby altering the bacterial microenvironment and exerting bactericidal effects. The prominent examples include gallium and cerium 151. Chen *et al.* 128 devised a multifaceted injectable composite hydrogel by integrating cerium-containing bioglass (Ce-BG) into a GelMA hydrogel. Ce-BG was synthesised via a fusion of sol-gel and template methods, effectively preserving the spherical morphology, chemical structure, and phase composition of the bioglass. The Ce-BG/GelMA hydrogel showed commendable cellular compatibility and facilitated the migration and tube formation of endothelial cells via Si ion release. *In vitro* antimicrobial assays revealed exceptional antibacterial attributes of the CeO2-incorporated bioglass/GelMA (5/G) composite hydrogel, particularly with 5 mol % CeO2 content. *In vivo* investigations revealed that the 5/G hydrogel significantly expedited wound healing in diabetic rats by accelerating granulation tissue formation, collagen deposition, and angiogenesis. Collectively, these findings underscore the therapeutic potential of the 5/G hydrogel in ameliorating diabetic wound healing.

### Cationic group

**Figure 10:**
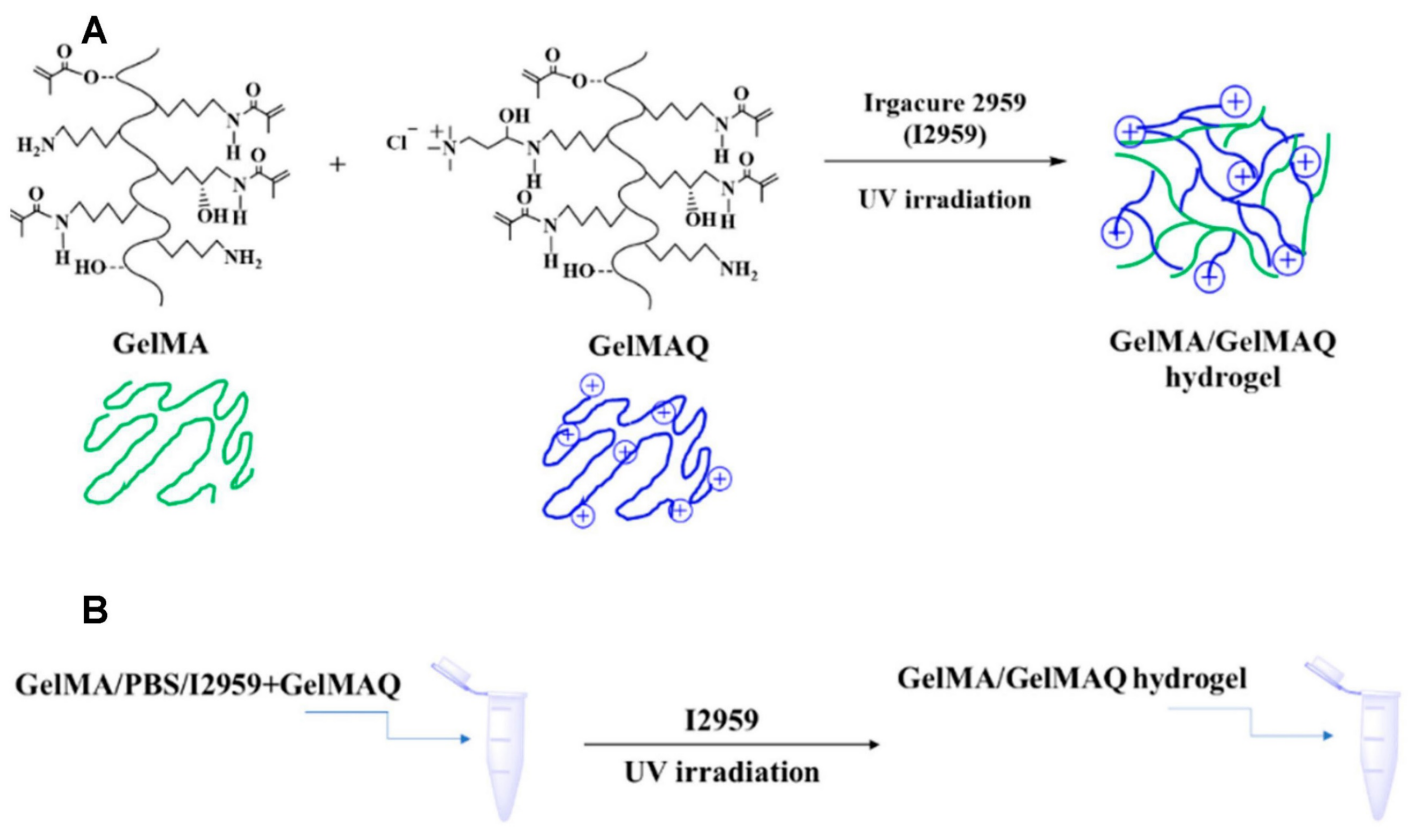
GelMA/GelMAQ hydrogel synthesis. (A) Synthetic chemical route for the GelMA/GelMAQ hydrogel synthesis, using I2959 as a crosslinker under UV irradiation. (B) General experimental procedure to GelMA/GelMAQ hydrogel synthesis. Under Creative Commons Attribution https://creativecommons.org/licenses/by/4.0/ Copyright 2023.

Cationic polymers with positive charges play a pivotal role in the treatment of bacterial infections and various other biomedical applications 152. They have a remarkable potential to combat antibiotic resistance and impede the formation of bacterial biofilms. Cationic polymers can proficiently dismantle preformed biofilms, and amphiphilic cationic polymers possess the capacity to self-assemble into nanoparticles capable of infiltrating biofilms, thereby enabling the polymers to eradicate both the biofilm and the bacteria residing within them. Vargas-Alfredo *et al.* 129 delved into the enhancement of GelMA through the incorporation of functional groups with innate antimicrobial properties. They synthesised GelMAQ by introducing modest degrees of substitution of methylacrylamide groups (DSMA) into GelMA and grafting glycidyltrimethylammonium chloride onto the free amino groups of the lysine residues in the original gelatine. Subsequently, they amalgamated GelMA with modified polymers containing elevated DSMA and GelMAQ at ratios of 50/50% or 25/75% (w/w) and juxtaposed them with the control groups of GelMA and GelMA. Chlorhexidine (CHX) was incorporated at a ratio of 0.2%. Various hydrogels were characterized using ^1^H-NMR spectroscopy and swelling behaviour assessments. These hydrogels were then scrutinised against *P. gingivalis* to gauge their antimicrobial properties, and their biocompatibility and regenerative attributes in human gingival fibroblasts were evaluated. In wound healing assays, GelMA/GelMAQ 25/75% exhibited commendable antimicrobial efficacy while demonstrating exceptional biocompatibility and regenerative potential in human fibroblasts. These findings offer novel insights into the development of hydrogels with antimicrobial and regenerative properties (Figure 10).

### Antibacterial methods based on photothermal therapy

Recently, PTT has garnered widespread attention for its efficacy in addressing bacterial infections owing to its minimal side effects, precise controllability, minimal invasiveness, high selectivity, and resistance to fostering multidrug resistance. Furthermore, research has indicated that photothermal stimulation within the temperature range of 40-42 °C can stimulate bone regeneration, thereby conferring upon PTT a distinctive advantage in treating infectious bone defects 153. Photothermal agents (PTAs) serve as the cornerstone of PTT, and the judicious selection of an appropriate PTA is pivotal for optimising treatment outcomes. Amid the escalating demand for antimicrobial interventions and the continual advancement of photothermal technology, PTAs have garnered considerable attention. In such systems, the heat generated by the photothermal effect not only inhibits bacterial proliferation, but also fosters tissue regeneration, thus offering dual therapeutic benefits. In the medical field, primary PTAs utilise both inorganic (IPTA) and organic (OPTA) types. IPTAs include metals such as silver (Ag), gold (Au), and copper (Cu), as well as carbon nanoparticles such as graphene oxide (GO) and carbon nanotubes (CNTs). OPTAs chiefly encompass organic dyes (such as Prussian blue [PB] and indocyanine green [ICG]) and polymer nanoparticles (e.g., polypyrrole [Ppy] and polydopamine [PDA]), the majority of which demonstrate commendable degradability and biocompatibility 153.

#### Inorganic photothermal agents

Metal nanoparticles are favoured for PTT owing to their cost-effectiveness, remarkable photothermal absorption properties, and broad-spectrum antimicrobial efficacy. Recently, considerable attention has been drawn towards composites that integrate metal nanoparticles into hydrogels. These composites exhibited robust stability and maintained an exceptional photothermal performance under specific light conditions. Qiu *et al.* 130 developed a composite hydrogel incorporating SrCuSi4O10 (SC), a microscale biomimetic ceramic composed of multilayer assembled nanosheets, in conjunction with GelMA. The SC/Gel composite hydrogel displayed proficient near-infrared photothermal conversion capabilities and diverse biological activities attributable to the sustained release of Sr2+, Cu2+, and SiO32- ions. Engineered for the treatment of infected dental pulp, this hydrogel effectively eradicated *S. mutans* and *L. casei* while impeding biofilm formation under photothermal heating. Furthermore, SC ion extracts facilitated dentinogenesis in rat dental pulp stem cells and angiogenesis in human umbilical vein endothelial cells (HUVECs). The therapeutic efficacy of the SC/Gel composite hydrogel-mediated vital pulp therapy was corroborated in a rat dental pulp infection model, demonstrating superior dentin-pulp complex repair compared to the commercially available iRoot® BP Plus. He *et al.* 154 introduced a PAG-CuS hydrogel, a polymer material derived from the copolymerisation of acrylic acid (AA) and GelMA modified with methacryloyl chloride, with a sodium lauroyl sulphate (LAS) coating on copper sulphide nanoparticles (CuS@LAS). This hydrogel has a porous three-dimensional structure conducive to cell adhesion and possesses remarkable water retention capabilities. The presence of CuS@LAS within the hydrogel not only confers photothermal antibacterial properties, but also serves as a physical crosslinker, augmenting its mechanical robustness. Upon near-infrared light-induced photothermal effects, the porous hydrogel released CuS@LAS, which subsequently decomposed via micelle disintegration to neutralise intracellular reactive oxygen species (ROS). This process culminates in the downregulation of MMP-9, which facilitates ECM synthesis and accelerates wound healing. Additionally, released Cu2+ from the PAG-CuS hydrogel augmented CD31 expression in endothelial cells, fostering microvascular formation, which is crucial for wound healing. In a diabetic wound model in GK rats, the application of the PAG-CuS hydrogel significantly mitigated ROS levels, augmented microvascular density, promoted epithelialisation, and expedited wound healing. Hence, this multifaceted photothermal hydrogel has the potential for sterilisation, free radical scavenging, and promotion of angiogenesis, rendering it a potent and comprehensive solution for managing the challenges posed by diabetic wounds.

#### Organic photothermal agents

**Figure 11:**
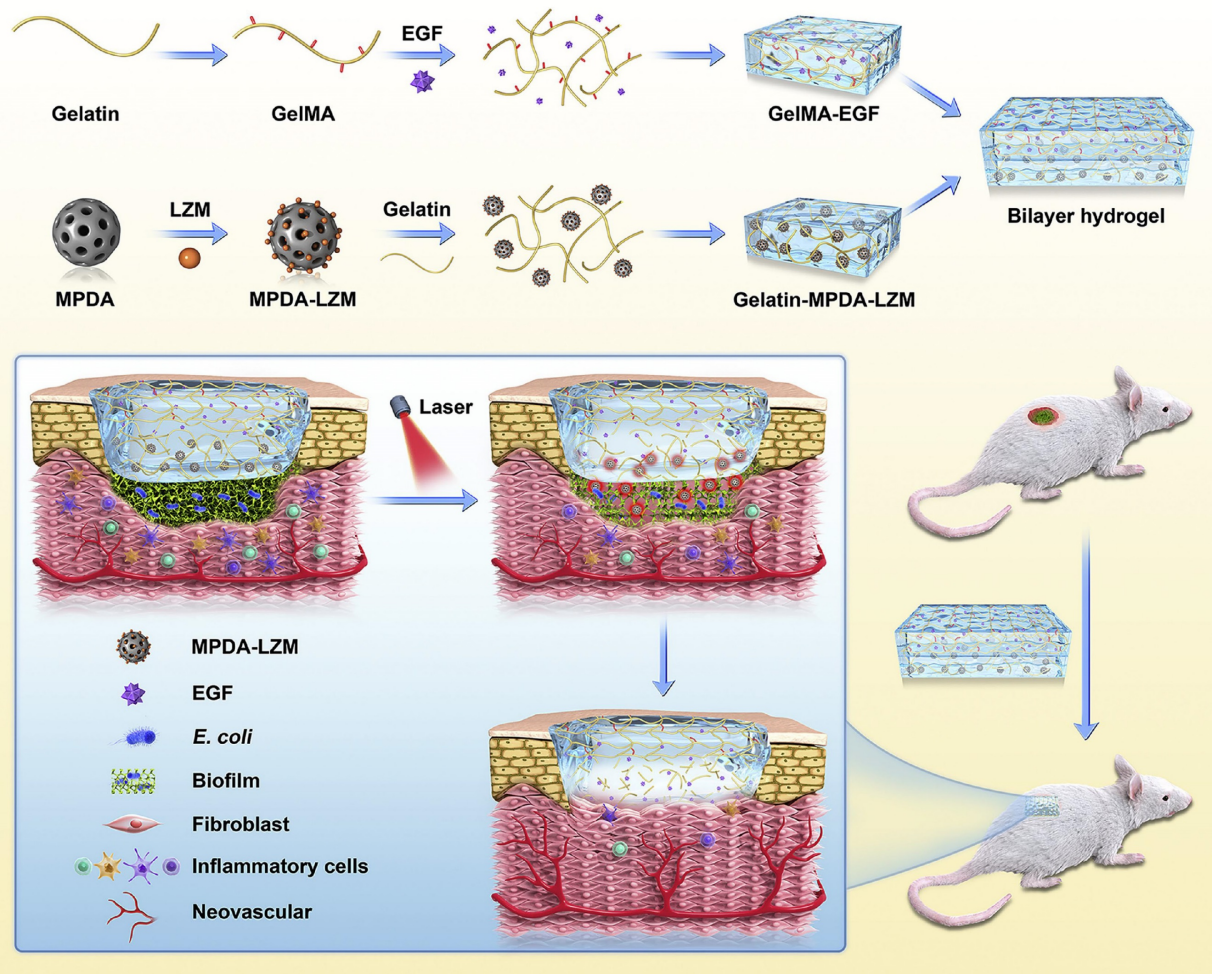
The preparation process of GelMA-EGF/Gelatin-MPDA-LZM bilayer hydrogel dressing and its application on chronic wounds. Reproduced from reference 135 Copyright 2022, with permission from Elsevier.

OPTAs predominantly encompass organic dyes such as ICG and PB, as well as polymer nanoparticles such as PDA and Ppy 153. Most OPTAs exhibit excellent biocompatibility and degradability. Despite the therapeutic promise of PTT under certain conditions, its efficacy is constrained by potential tissue damage from excessive heat and logistical hurdles in the storage and delivery of PTAs, which hinder the complete eradication of biofilms via PTT. To mitigate these challenges, Wang *et al.* 134 introduced a pioneering GelMA-EGF/gelatine-MPDA-LZM dual-layer hydrogel dressing engineered to combat biofilms through PTT bolstered by lysozyme (LZM) while concurrently expediting the healing of chronic wounds. Within this dual-layer hydrogel system, gelatine forms the inner layer, tasked with housing mesoporous polydopamine (MPDA) nanoparticles (MPDA-LZM) laden with lysozyme. Upon temperature elevation, these nanoparticles swiftly liquefy, facilitating substantial release. MPDA-LZM nanoparticles not only function as PTAs with antimicrobial properties but also penetrate and disrupt biofilms. The outer hydrogel layer is comprised of GelMA and EGF, which foster wound healing and tissue rejuvenation. This dual-layer hydrogel dressing exhibits notable anti-infective properties and accelerates wound healing *in vivo*. Furthermore, nerve innervation plays a pivotal role in bone regeneration, serving as both an instigating factor and a crucial regulator of subsequent processes such as angiogenesis, ossification, and mineralisation. Hence, this innovative dual-layer hydrogel dressing shows promise for advancing bone regeneration and repair (Figure 11).

## Infectious bone defects treatment strategies

### Smart stimuli-responsive GelMA-based strategies

Polymer hydrogels are composed of water-soluble polymers that form a flexible three-dimensional crosslinked network through appropriate crosslinking, resulting in a multiphasic system with water. This system responds accordingly when subjected to environmental stimuli, classifying it as an intelligent polymer material or smart hydrogel 155. These hydrogels exhibit sensitive responses to external stimuli. In contrast to traditional hydrogels, stimulus-responsive hydrogels demonstrate sensitivity in both spatial and temporal dimensions, enabling products made from these materials to possess multiple, adjustable, and controllable characteristics. The introduction of specific responsive functional groups combined with natural polymers is a key approach to imparting stimulus responsiveness to hydrogels 156. This section provides an overview of the response strategies of GelMA hydrogels to various stimuli, including those that address internal pathological microenvironmental stimuli (such as pH, ROS, enzymes, and glucose levels) and external physical stimuli (such as thermal, electrical, optical, magnetic, and mechanical forces).

#### The pH- response strategies

**Figure 12:**
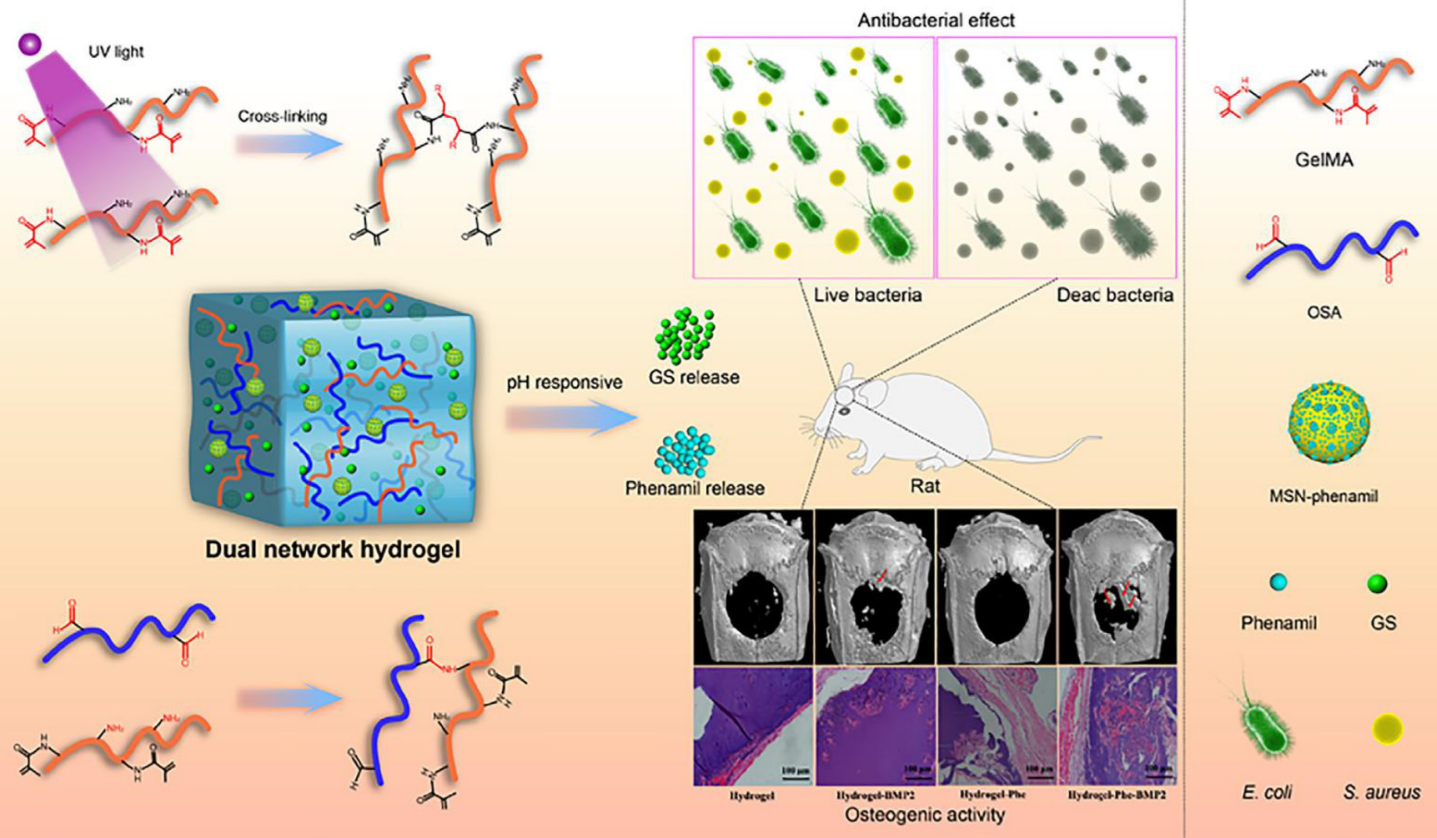
Schematic diagram of GelMA-OSA hydrogels preparation and fast large bone defect repair. Reproduced from reference 160 Copyright 2022, with permission from Elsevier 144.

The normal microenvironment of the human body is weakly alkaline with a pH of 7.4 157. In the presence of inflammation, infection, or tumours, the pH of affected tissues can be 0.5 to 1 unit lower than that of surrounding healthy tissues 158. This mildly acidic microenvironment can be exploited to develop GelMA-based reactive biomaterials that promote bone healing and regeneration. Yao *et al.* 159 introduced oxidised sodium alginate (OSA) into GelMA to create pH-responsive double-network GelMA-OSA hydrogel scaffolds. In these scaffolds, light energy is converted into gentle heating (40°C - 43°C), leading to a reaction between the aldehyde groups in OSA and the amine groups in GelMA, resulting in the formation of Schiff base linkages (-C=N-). Under acidic conditions associated with bacterial infection, the Schiff base linkages in the GelMA-OSA hydrogel break, releasing gentamicin sulphate (GS, an antibacterial agent) and phenylalanine (Phe, a small-molecule activator of BMP-2) encapsulated within the GelMA, thereby exerting antibacterial and osteogenic effects. Additionally, ferritin possesses pH-dependent assembly characteristics, allowing it to be loaded with various substances (such as drugs and DNA) and release them in response to specific pH changes, thereby achieving a pH-responsive targeted drug delivery system (Figure 12).

#### Reactive oxygen species-response strategies

**Figure 13:**
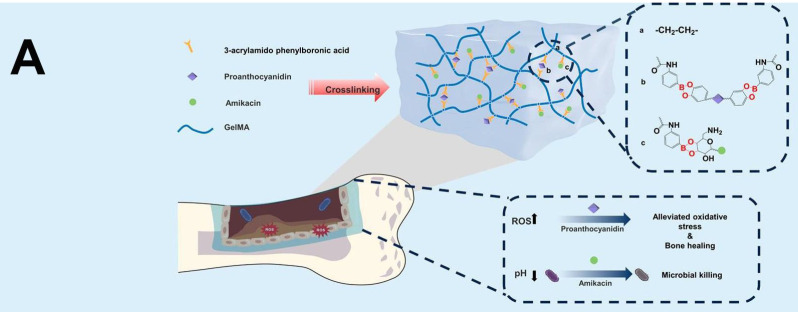
Schematic diagram of GelMA-OSA hydrogels preparation and fast large bone defect repair. (A) smat hydrogel delivery system for dual delivery of proanthocyanidin and amikacin at bone defect interfaces based on the unigue pH and Ros responsiveness of boronate complexes; (B) The hydrogel can timely respond to changes in pH and Ros levels of the bone defect microenvironment, release proanthocyanidin and amikacin on demand to respectively cope with the inflammation and infection problems in the early stage, thus promoting the early healing of bone tissue. Reproduced from reference 160 Copyright 2022, with permission from Elsevier.

ROS are a group of substances generated through the one-electron reduction of oxygen, including superoxide anions, hydrogen peroxide, hydroxyl radicals, and nitric oxide. They play vital roles in various biological processes including cellular signal transduction and metabolism 156. However, excessive ROS production can lead to oxidative stress, resulting in the degradation of ECM mineralisation and abnormal bone metabolism 156. Consequently, the development of reactive biomaterials with the capacity to scavenge ROS is crucial for the treatment of bone diseases and regenerative medicine. Bacterial infections can trigger inflammatory responses, leading to the accumulation of ROS and a reduction in local pH levels. In an acidic environment, osteoblast activity is inhibited, posing significant challenges to the repair and regeneration of bone defects. To address this complex microenvironment, researchers such as Qi *et al.* 160 utilised the unique pH and ROS responsiveness of borate complexes and combined them with GelMA to successfully synthesise a novel smart GelMA hydrogel. This hydrogel was capable of simultaneously delivering two drugs, procainamide and amikacin. The incorporation of the borate complexes improved the mechanical properties, swelling rate, degradation kinetics, and antioxidant capacity of the hydrogel system. Most notably, this hydrogel exhibited dual sensitivity to pH and ROS, enabling the coordinated regulation of drug release. They reported that during the release of proanthocyanidins, the hydrogel responds to changes in ROS levels, thereby protecting mouse osteoblast precursor cells from oxidative stress damage and promoting their differentiation into osteoblasts. Additionally, this hydrogel sensed fluctuations in pH, releasing an appropriate amount of amikacin in a timely manner to exert an effective antibacterial action (Figure 13).

#### Enzyme-response strategies

Enzymes are a class of highly specific and selective biomolecules that play crucial roles in biological processes such as bone growth, resorption, and onset and progression of bone diseases 161. By exploiting these properties, researchers have developed GelMA hydrogels with intelligent response characteristics that can act as biological triggers. MMPs are a family of enzymes primarily involved in ECM remodelling of the ECM 162. Studies have shown that the expression levels of MMPs are abnormally elevated in diseases such as osteoarthritis and osteoporosis. The presence of MMP recognition sequences within the GelMA molecules allows for their degradation in environments with high MMP levels. These characteristics render GelMA an ideal material for the development of MMP-responsive drug delivery systems. Ribeiro *et al.* 163 synthesised a GelMA-based hydrogel that released chlorhexidine (CHX) on demand. When exposed to elevated levels of MMPs, the GelMA hydrogel released CHX, thereby exhibiting antibacterial properties. However, to date, no studies have combined MMP-responsive GelMA hydrogels with osteogenic drugs or growth factors. Therefore, the potential applications of MMP-responsive GelMA hydrogels in the treatment of osteoarthritis and bone tumours and promotion of bone formation require further investigation. The development of such intelligent hydrogel systems not only aids in disease treatment but may also provide new strategies for personalised medicine.

#### Photo-responsive strategies

**Figure 14:**
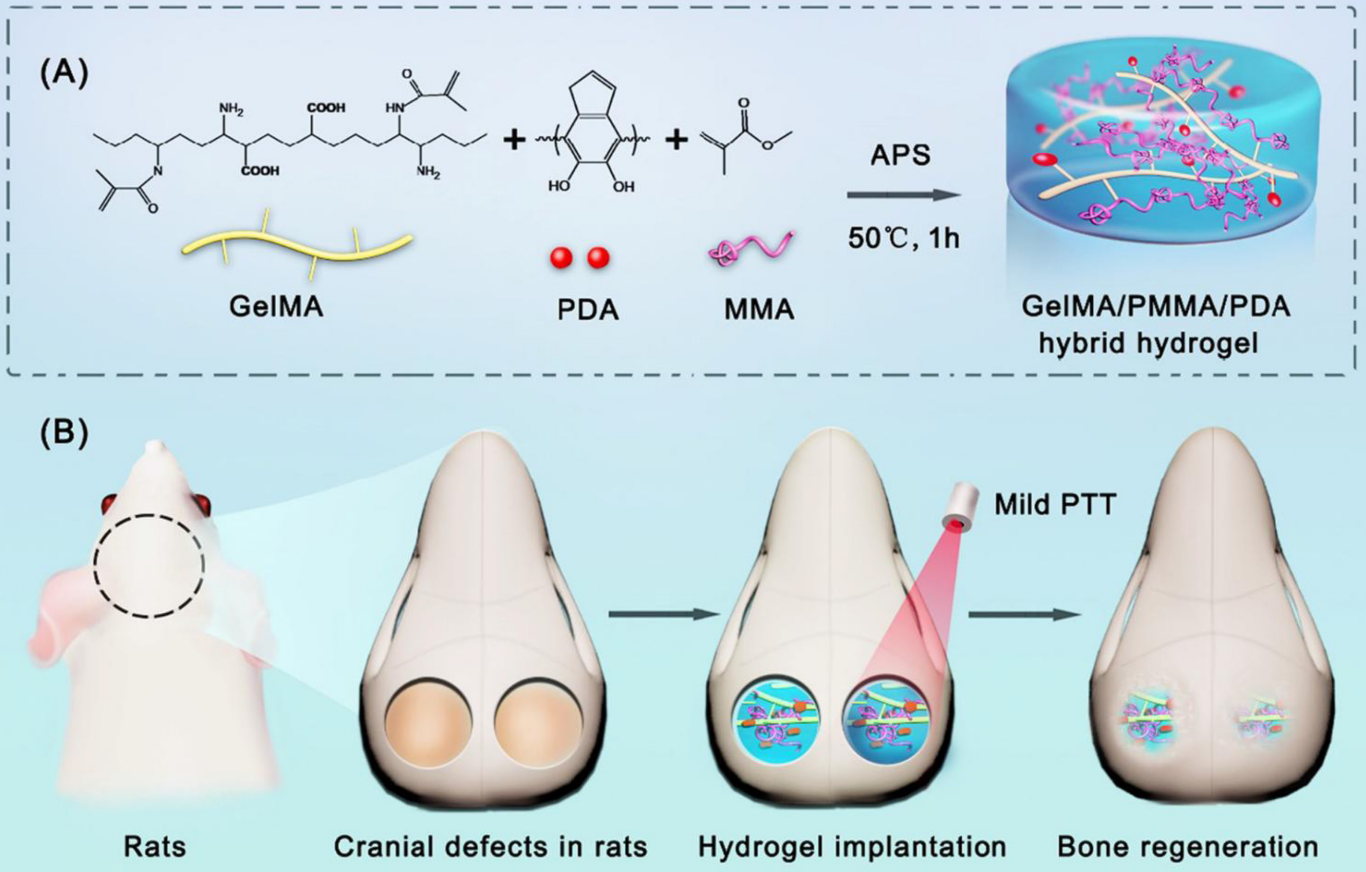
(A) Preparation of GelMA/PMMA/PDA hydrogel. (B) Illustration of GelMA/PMMA/PDA hydrogel with mild PTT for skull regeneration. Reproduced from reference 165 Copyright 2022, with permission from Elsevier.

PTT utilises PTAs to convert light energy into mild heat (40°C - 43°C), demonstrating significant application potential in the field of bone regeneration 164. Based on this principle, researchers led by Wu *et al.* 165 successfully developed a GelMA/poly(methyl methacrylate) (PMMA)/polysaccharide amine nanoparticle (PDA) light-responsive hydrogel. PDA serves as a photothermal conversion agent, effectively transforming light energy into thermal energy under 808 nm laser irradiation. The results indicated that this composite light-responsive hydrogel exhibited excellent osteogenic effects in both the *in vitro* and *in vivo* calvarial defect repair experiments (Figure 14). In another study, Nie *et al.* 60 developed a composite biolink consisting of GelMA/β-tricalcium phosphate (β-TCP)/sodium alginate (Sr2+)/MXene (Ti3C2) (GTAM). The incorporation of MXene endowed this biolink with remarkable photothermal properties, enabling it to generate heat under near-infrared light irradiation, thereby effectively eradicating bacteria. In addition, GTAM biolink promotes osteogenic differentiation. Ultimately, this dual-functional bio-link successfully facilitated the regeneration of infected mandibular defects within bioprinted scaffolds loaded with bone marrow mesenchymal stem cells. These studies highlight the broad application prospects of integrating PTT with biomaterials in bone regenerative medicine.

#### Temperature-responsive strategies

Among the various external stimuli, temperature changes are considered an easy-to-use and relatively safe source of stimulation. Temperature-sensitive hydrogels have been widely studied in the field of smart hydrogels. For thermosensitive natural polymer gels, the rapid phase transition near the lower critical solution temperature (LCST) significantly influences the gel state, which has close relevance and broad application potential in medicine and pharmaceuticals. Researchers led by Luo 166 developed a thermo/optically double-crosslinked hydrogel using GelMA and methylmethacrylate-based chitosan (MHBC). As a thermosensitive material, MHBC enables hydrogels to shrink uniformly and reversibly with increasing temperature. GelMA provides excellent biocompatibility to composite hydrogels and enhances cell adhesion. Future research directions for GelMA temperature-responsive hydrogels will focus on achieving precise temperature regulation and flexible temperature control technologies. Although there are limited studies on the application of GelMA hydrogels with temperature-responsive strategies for infectious bone regeneration, these investigations lay the groundwork for future research. It is anticipated that the GelMA composite hydrogels with temperature-responsive properties will selectively release therapeutic drugs or promote active osteogenic molecules in a time- and space-controlled manner, leading to improved treatment outcomes for bone diseases and enhanced bone regeneration.

#### Electrical response strategies

Polymer electrolyte gels and some composite gels doped with electrical materials exhibit specific responsiveness to electric fields. When hydrogels are placed in an external electric field, they undergo contraction or expansion, accompanied by the conversion of electrical energy into mechanical energy. This characteristic endows hydrogels with multiple functionalities, showing promising application prospects in sensors, controlled drug release, and artificial muscles within the fields of medicine and smart materials. By combining electroactive materials with biomaterials, it is possible to produce "smart" biomaterials that respond to electrical stimulation, enabling more precise regulation of cellular activities and drug release, thereby achieving better bone regeneration and therapeutic effects 167. Although GelMA hydrogels lack electrical conductivity, they can be combined with conductive polymers such as polypyrrole (PPY) 74 and polyaniline (PANI) 168 to impart responsiveness to electrical stimulation. Dutta *et al.* 74 introduced Fe ions into GelMA grafted with PPY, adjusting the conductivity of the bio-link to levels comparable to that of cortical bone (approximately 0.2 mS cm^-1^) and trabecular bone (approximately 0.79 mS cm^-1^). Piezoelectric hydrogels constitute another category of electrically responsive biomaterials. The piezoelectric effect allows them to convert mechanical forces into electrical stimuli, making them particularly suitable for bone defects in pressurised areas, such as the alveolar and limb bones. Liu *et al.* 169 fabricated a nanocomposite hydrogel composed of piezoelectric quadrilateral BaTiO₃ nanoparticles and GelMA. Under mechanical stimulation, this hydrogel can generate electrical stimulation, improving the mitochondrial bioenergetic function in periodontal ligament stem cells (PDLSCs) and promoting their osteogenic differentiation.

Although the application of electrically responsive GelMA biomaterials in infectious bone regeneration is currently limited, the aforementioned studies indicate significant application potential for utilizing the electrical stimulation-responsive GelMA in the field of bone regeneration, with expectations for more breakthroughs in the near future.

### 3D‑Bioprinted GelMA‑Based Strategies

Traditional methods for fabricating tissue engineering scaffolds include porogen (or template) methods, phase separation, gas foaming, and freeze-drying 170. With the continuous development of clinical requirements and scientific research, these methods face three main challenges: 1. difficulty in flexibly designing and precisely controlling the microscopic structure of scaffolds; 2. difficulty in manufacturing scaffolds with shapes that closely match the injured site; and 3. difficulty in constructing scaffolds with heterogeneous features. Additive manufacturing (3D printing), a computer-assisted forming technique, has the advantage of accurately and rapidly constructing the macromorphology and microstructure of scaffolds, enabling personalised tissue repair and regeneration 38. The focus of 3D printing research has been on the development of bioinks composed of biomaterials, cells, and functional factors. In addition to traditional requirements such as biocompatibility, mechanical strength, and biodegradability, 3D printing inks must also possess printing adaptability (including structural fidelity, structural resolution, and cell viability) 38. The rapid gelation, photopolymerization characteristics, and biocompatibility of GelMA meet the requirements of the widely used 3D printing technologies. After the bioprinting solution is extruded from the machine, it undergoes focused UV radiation for an appropriate time, leading to rapid curing under UV light at the desired location 171. Therefore, owing to its excellent plasticity and ability to conform to defects, GelMA is often combined with 3D printing technology for bone tissue engineering applications to construct scaffolds for more complex bone defect repairs.

Currently, many 3D printing materials for bone repair are prepared using extrusion-based bioprinting methods, which have significant limitations in terms of structural integrity and manufacturing speed for bone substitutes. Tao *et al.* reported the encapsulation of rBMSCs in GelMA/dextran emulsions using a DLP-based 3D bioprinting platform 59. DLP is a surface projection-based bioprinting method that offers higher printing resolution and reproducibility than the widely used extrusion printing method. Unlike the "boundaries" formed between droplets in inkjet bioprinting or between adjacent fibres in extrusion printing, DLP technology allows for smoother stacking of three-dimensional structures, significantly enhancing structural integrity and mechanical performance. This 3D hydrogel scaffold has been shown to promote the proliferation, migration, diffusion, and osteogenic differentiation of rBMSCs by modulating the YAP signalling pathway. Furthermore, in-vivo experiments on rat cranial defects have confirmed the therapeutic effects of these scaffolds.

**Figure 15:**
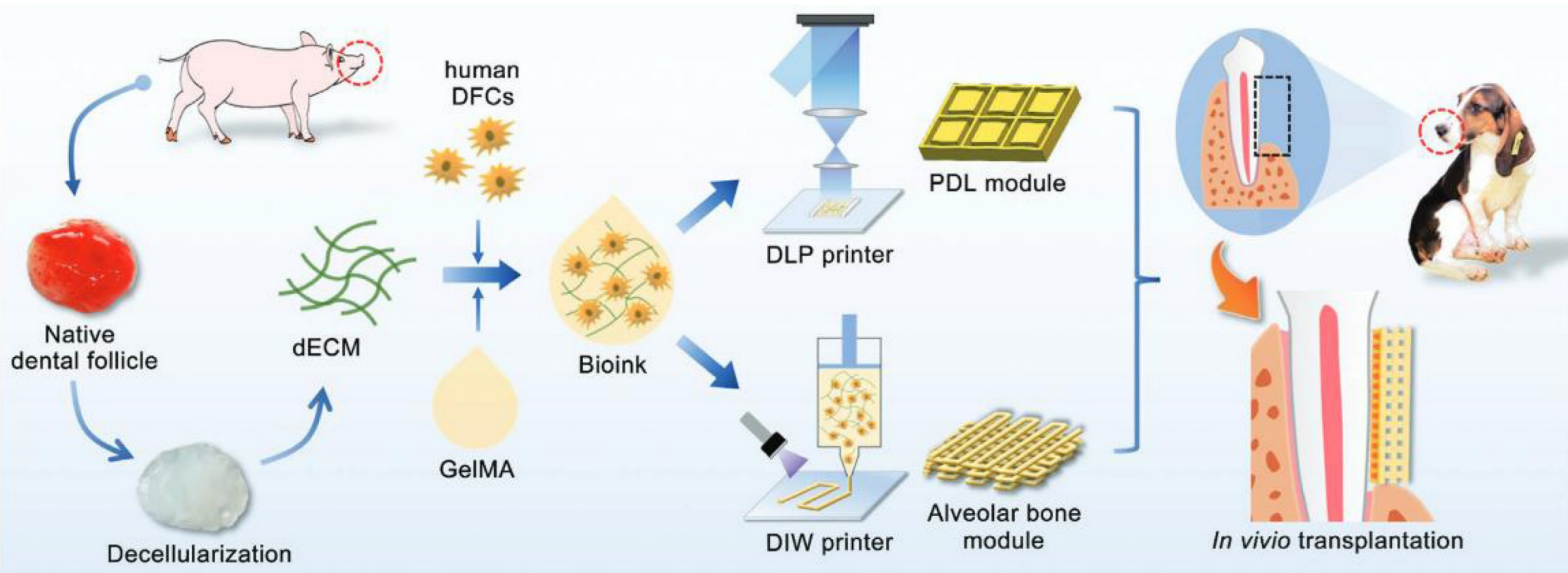
Schematic overview of the 3D bioprinting of periodontal modules with GelMA/dECM bioink encapsulating human DFCs for periodontal regeneration. Under Creative Commons Attribution https://creativecommons.org/licenses/by/4.0/ Copyright 2022.

In addition to customising scaffolds for specific bone defect shapes using bioprinting technology, it is essential to focus on the biological functions of printed structures. There have also been attempts to use decellularised matrix hydrogel bioinks. For instance, Yang *et al.* 172 combined a decellularised ECM (dECM) from pig hair follicles with GelMA to create bioinks. The addition of the dECM provided a supportive microenvironment for the loaded periodontal stem cells, promoting periodontal tissue regeneration (Figure 15). In a recent study, Gao *et al.* 20 incorporated decellularised pig bone powder into GelMA for use as a bone tissue engineering scaffold. They demonstrated that the scaffolds could induce the osteogenic differentiation of human bone marrow mesenchymal stem cells, even without induced media.

Compared to allogeneic growth factors, autologous growth factors have significant advantages such as high biosafety, low rejection rates, and excellent efficacy, although their sources are relatively limited. Researchers have attempted to use patient-specific biological cues to promote bone regeneration in printed structures. Patient-specific biological cues offer clear advantages, including easy accessibility, avoidance of immune rejection, and reduced ethical regulatory burden in clinical translation. Platelet-rich plasma (PRP) and patient-derived bone particles are currently used to prepare patient-specific bioinks based on GelMA hydrogels. Patient-derived PRP provides abundant autologous growth factors for printed structures, thereby promoting stem cell recruitment, osteogenesis, and vascular formation 173. The presence of bone particles not only enhances the mechanical properties of printed structures but also provides viable cells and forms a complete mineralised matrix, all of which are beneficial for osteogenesis 174.

## Conclusion and prospective

This review describes the latest research progress on GelMA hydrogel-based biomaterials for the treatment of infectious bone defects. First, we briefly introduced the main types of biological materials, along with their advantages and disadvantages. We focused on the synthesis and properties of GelMA hydrogels, followed by a summary of their applications in promoting osteogenesis and exhibiting antibacterial activity. Finally, we discussed the application of GelMA hydrogels for the treatment of infectious bone defects.

Although significant strides have been made in the development of GelMA-based hydrogels, challenges and unresolved issues remain. Future research directions include:

1. Source, Synthesis, and Safety of GelMA: Currently, there is no standardised framework for the origin, synthesis, or safety of GelMA, making it a valuable area of study.

2. Biodegradation and Stability: GelMA hydrogels suffer from rapid biodegradation and lack long-term stability, resulting in performance loss. Addressing this issue will require adjusting material formulations and improving processing techniques. For example, adding more biodegradable materials, optimising crosslinking, and introducing stabilisers can enhance the biocompatibility, stability, and photothermal effects 11. Therefore, selecting suitable materials for specific applications is crucial, as addressing these issues is a prerequisite for advancing their clinical application. For instance, a company modified GelMA with silk fibroin to create a new product, Silk Fibroin Methacryloyl (SilMA), which combines the excellent biocompatibility, stable degradation performance, and osteogenic properties of silk fibroin. This product has garnered significant attention owing to its ability to dissolve quickly in water and to photocrosslink into hydrogels 175,176.

3. Cost and Technical Sensitivity: While materials may possess ideal properties, they can be technically sensitive and costly, limiting their feasibility for large-scale production and applications. For example, AgNPs, although highly effective with broad-spectrum antibacterial activity, are relatively expensive and prone to oxidation at room temperature. Many researchers are drawing inspiration from plant-based materials such as A. vera and curcumin, which offer broad availability and low costs. Therefore, optimising production, reducing costs, and ensuring scalability of materials are essential for their widespread use 16,22

Several promising approaches are worth considering to further optimise construction strategies for GelMA hydrogels:

1. Smart Responsive Antibacterial and Osteogenic Hydrogels: Traditional assembly methods have limitations. Developing microenvironment-responsive materials that possess biodegradability, drug-loading capacity, and anti-infective and anti-inflammatory properties aligns better with clinical needs. To date, no studies have combined GelMA hydrogels with stimuli-responsive mechanisms such as glucose, enzymes, or magnetic fields for infectious bone defects, making this a novel and valuable avenue of research.

2. Piezoelectric Antibacterial Osteogenic Hydrogels: Recently, there has been a growing awareness of the importance of bioelectric communication between cells. Piezoelectric materials are gaining attention because of their ability to generate electrical stimuli under stress, mimic the bioelectric microenvironment of bone tissue, and enhance osteogenic bioactivity 177. GelMA hydrogels combined with conductive materials offer a promising route for creating multifunctional tissue structures with biocompatibility and piezoelectric properties. Hybrid hydrogels with piezoelectricity and low toxicity represent the next-generation bone tissue engineering scaffolds for treating bone defects.

3. Carbon Quantum Dot (CQD) Antibacterial Osteogenic Hydrogels: CQDs are carbon particles with diameters between 1-10 nm that exhibit fluorescent properties. They have two main advantages: their unique fluorescence makes them ideal for bioimaging, and their simple, environmentally friendly synthesis makes them highly accessible 178. Researchers have developed p-CQD/WS2 composites and p-CQD/WS2/n-CQD-coated GelMA hydrogels that effectively kill multi-drug resistant bacteria and promote bone regeneration, making them promising solutions for repairing infectious bone defects 132.

In summary, as bone tissue engineering continues to advance rapidly, it is crucial to address the challenges related to its mechanical performance, degradation behaviour, microstructure, bioactivity, and long-term biocompatibility. Despite these challenges, continuous progress in GelMA-based bone tissue engineering technologies suggests that GelMA hydrogel substitutes have great potential for the clinical treatment of infectious bone defects in the near future.

## Figures and Tables

**Table 1:** Advantages and disadvantages of different source materials.

Hydrogel materials	Advantages	Limitations	Application
natural hydrogel			
collagen	good biocompatibility and biodegradability; good tensile strength; mimics natural extracellular matrix (ECM) of dentin 26	poor mechanical strength 27	soft tissue engineering, bone tissue regeneration, and wound healing 28
chitosan	nontoxic and easily bioabsorbable; antibacterial activity 29	poor mechanical performance; poor control over hydrogel pore size 29	wound dressings, drug delivery systems, and antibacterial materials 29.
silk	good biocompatibility; biodegradability; oxygen and water permeability; morphologic flexibility 30	poor degradation performance 30	bone tissue engineering, vascular repair, nerve regeneration, and wound healing 30
alginate	nontoxic; good biocompatibility 31	mechanically weak 31	tissue engineering scaffolds, cell encapsulation and transplantation, and drug delivery 31
chondroitin sulphate	biocompatible; biodegradable; bioactive; nonimmunogenic 32	water soluble; low mechanical integrity 32	soft tissue repair, plastic and cosmetic surgery, drug delivery systems 28
synthetic hydrogel			
polylactic acid (PLA), poly‑l‑lactic acid (PLLA), polyglycolic acid (PGA), PLGA, and polyεcaprolactone (PCL)	nontoxic and biodegradable 33	chronic or acute inflammatory 33	load-bearing bone scaffolds, soft tissue repair, controlled drug release systems 33
hybrid hydrogel			
PVA-SA	good mechanical strength; multifunctionality 34	poor biodegradability; Complex fabrication; Potential toxicity 34	drug delivery systems; Tissue engineering scaffolds 24
PVP-HA	good mechanical strength; multifunctionality 34	poor biodegradability; Complex fabrication; Potential toxicity 34	drug delivery systems; Tissue engineering scaffolds 25

**Table 2:** Summary of typical GelMA-based scaffolds for accelerating bone regeneration.

GelMA-based bone scaffolds with cells						
GelMA hydrogel scaffolds	Cells lines	Physical properties	Bioactivities	Mechanism		
bio-GelMA 57	BMSC	injectability; porous structure	good cytocompatibility and proliferative properties, promoted new bone formation and angiogenesis			
GelMA-HAP-SN 58	BMSC	injectability	excellent injectability, cell activity, and osteogenesis			
GelMA)/dextran emulsion 59	BMSC	porous structure, good mechanical and degradation properties	good proliferation, migration, spreading, and osteogenesis	YAP signal pathway		
GelMA/β-TCP/Alginate/MXene 60	rBMSCs, RAW264.7	excellent shear-thinning properties, and suitable viscosity, good mechanical strength	good biocompatibility, excellent antibacterial properties, promoted healing of infected bone defects			
GelMA-RF 61	KUSA-A1	delayed photocrosslink curing and suitable mechanical function	higher cell viability, promoted osteoblast differentiation			
GelMA-BMSCs/PLA-PGA-PLA-ECs 62	BMSCs, RAOECs	sufficient mechanical properties and good permeability	increased cell viability, promoted differentiation and maturation of osteoblast			
GelMA/HAMA/Alginate/GO 63	BMSCs, BMMs	stable porous structure, suitable mechanical, swelling and degradation properties	promoted polarization of BMMS to M2 type, promoted osteogenic differentiation of BMSCs, improved osteogenic repair			
GelMA/HAMA/DBM/VEGF 64	BMSCs	high mechanical strength, appropriate biodegradation rate and controllable VEGF release	biocompatibility, excellent ectopic bone regeneration ability			
Eth-DFO@GelMA/GGMA 65	BMSCs, HUVECs	improved printability and mechanical property, slow release of DFO	promoted migration and tube formation of ECs, improved osteogenesis and angio- genesis			
GelMA/OMP 66	hMSCs	enhanced mechanical properties, prolonged oxygen release	enhanced mechanical properties, prolonged oxygen release			
GelMA/SiGO 67	hMSCs	enhanced production, retention and bioactivity of BMPs	improved mineralization and accelerated bone repair	BMP-SMAD1/5 signalling pathway		
GelMA-Alg-WH/HAP-hMSCs 68	HUVECs, hMSCs	good mechanical, swelling, degradability properties	good cytocompatibility and excellent osteogenic properties			
EphrinB2-DPSC-GelMA 69	DPSCs	good mechanical properties	improved mineralization and accelerated bone repair			
GelMA-based bone scaffolds with growth factors and their substitutes						
GelMAhydrogel scaffolds	growth factors and their substitutes	physical properties	bioactivities	mechanism		
GelMA-HAp-HAD/Col I 70	col I	excellent swelling properties, mechanical stability and delayed degradation	promoted the migration and differentiation of BMSCs, improved angiogenesis and bone regeneration			
Ti2448-GelMA/DFO 71	DFO	good osteoconductivity and osteointegration	improved the osteogenesis, angiogenesis for large bone defects			
MSNs-NH2@GelMA 72	pyritum (PP)	good mechanical properties	promoted the differentiation of BMSCs and improved the osteogenesis			
C/BCM-GelMA 73	BMP-2	rapid molecular release	inhibited the inflammatory response and improved the osteogenesis			
GelMA-PPy-Fe 74	BMP-2	excellent shape fidelity, enhanced conductivity	good cytocompatibility and improved osteo- genic differentiation	NOTCH/MAPK/SMAD signalling		
BMSCs-BMP2-GelMA 75	BMP-2	appropriate mechanical properties; injectability	promoted the differentiation of BMSCs and improved the osteogenesis			
BMSC/RAW/BMP-4-GelMA 76	BMP-4	good mechanical properties	improved anti-inflammatory and osteogenesis			
GelMA/HAMA-OGP 77	OGP	good mechanical properties and photocrosslink ability	improved the osteogenesis			
GelMa-PP&VEGF 78	VEGF	high mechanical strength, appropriate biodegradation rate and controllable VEGF release	biocompatibility, excellent ectopic bone regeneration ability			
GelMA/HAMA/DBM/VEGF 15	VEGF	high mechanical strength, appropriate biodegradation rate and controllable VEGF release	biocompatibility, excellent ectopic bone regeneration ability			
GelMA-Lip@VEGF 79	VEGF	controllable VEGF release	improved the osteogenesis			
F/G-B/M 80	BFGF and BMP-2	controllable VEGF and bFGF release	improved the osteogenesis and angiogenesis			
GelMA-PVA (GP) 81	PTH	controllable PTH release	improved the osteogenesis			
GelMA-based bone scaffolds with exosome						
GelMAhydrogel scaffolds	exosome	physical properties	bioactivities	mechanism		
hypo-ADSC-Exos-gel 82	ADSC-exos	controllable	improved of HUVEC proliferation, migration, and angiogenesis	PI3K/AKT signalling pathway		
ExoBMP2+NoBody 83	ExoBMP2	Delayed degradation	improved the osteogenesis			
ExoLnc NEAT1-alginate/GelMA 84	HUVEC-exos	mechanical stability and delayed degradation	improved bone regeneration, facilitate the angiogenesis, increase the infiltration of M2 polarized macrophages	DDX3X/NLRP3 axis		
USCEXOs/GelMA-HAMA/nHAP 85	USCEXOs	controllable and biocompatible	improved osteogenesis and angiogenesis			
BG-gel-sEVs 86	hUC-MSCs-sEVs	controllable and biocompatible	improved vascularized bone regeneration	PTEN/AKT signalling pathway		

**Table 3:** Summary of typical GelMA-based hydrogel scaffolds for controlling infected tissue.

GelMA-bases hydrogel	Metal ions	Bacteria	Application
GelMA/HA-E/Ag@MOF 120	EGCG	*E. coli* + *S. aureus*	Antibacterial effect, wound healing
GelMA-NP50-Cur3 121	cur	*E. coli* + *S. aureus*	Antibacterial effect, wound healing
Nio-Thymol@GelMa 122	Nio-Thymol	*E. coli* + *S. aureus*	Antibacterial effect, wound healing
GelMA-WEO 123	WEO	*E. coli* + *S. aureus* + MRSA	Antibacterial effect, wound healing
GelMA/AV 124	AV	*E. faecalis*	Endodontic disinfection and immunomodulation
GelMA-bases hydrogel	Metal ions	Bacteria	Application
PSBDA 125	Ag	*E. coli* + *S. aureus*	Antibacterial effect, wound healing
GelMA 126	ZF	*S. aureus*	Antibacterial effect, wound healing
GelMA 127	SPION	*S. aureus*	Antibacterial effect, wound healing
Ce-BG/GelMA 128	Ce	*E. coli* and *S. aureus*	Antibacterial effect, wound healing
GelMA-bases hydrogel	Cationic group	Bacteria	Application
GelMAQ 129	DSMA	*Porphyromonas gingivalis*	Periodontal tissue regeneration
GelMA-bases hydrogel	PAT	Bacteria	Application
SC/gel 130	SC	*Streptococcus mutans* + *Lactobacillus casei*	Antibacterial effect and enhanced pulp, regeneration activity
PAG-CuS 131	CuS	*E. coli* + *S. aureus*	Antibacterial effect, wound healing
p-CQD/WS2/n-CQD/GelMA 132	p-CQD/WS2	*E. coli* + *S. aureus*	Osteogenic and antibacterial effects
GelMA-PAM 133	MOF	*E. coli* + *S. aureus*	Antibacterial effect, wound healing
GelMA-EGF/ -MPDA-LZM 134	MPDA-LZM	*E. coli*	Osteogenic and antibacterial effects
GelMA-Au NBPs@SiO2 135	Au NBPs	*P. gingivalis*	Osteogenic and antibacterial effects
GelMA/HA-DA/GO-βCD-BNN6 136	GO	*E. coli* + *S. aureus*	Antibacterial effect, wound healing
ZPTA-G/HMA 137	ZnO@PDA	*E. coli* + *S. aureus* + *Candida albicans*	Antibacterial and anti-inflammatory effects
GelMA 138	BP@Mg	*E. coli* + *S. aureus*	Antibacterial effect and innerved bone regeneration
CuSNP@CS-MA 139	CuS	*E. coli* + *S. aureus*	Periodontal tissue regeneration

**Table 4:** Summary of GelMA-based hydrogel scaffolds for infectious bone regeneration

GelMA hydrogel scaffolds	Strategies	Bioactivities	Application
GPA hydrogel	ROS- response	promoting early-stage bone healing in infectious bone defect	targeted drug delivery systems 160
GelMA-OSA	PH-response	antibacterial and osteogenic agents	targeted drug delivery systems 159
GelMA-CHX	MMP-response	antibacterial	targeted drug delivery systems 163
GelMA-PMMA-PDA	photo-responsive	osteogenic	bone tissue engineering scaffolds 165
GTAM	photo-responsive	antibacterial and osteogenic agents	bone tissue engineering scaffolds 60
GelMA-MHBC	temperature response	promoting cell adhesion ability	cell culture 166
GelMA-BaTiO3	electrical response	promoting cell osteogenic differentiation	bone tissue engineering scaffolds 74
GelMA-dextran	DLP	promoting osteogenics	bone tissue engineering scaffolds 59
GelMA-dECM	3D-bioprinted	promoting periodontal tissue regeneration	bone tissue engineering scaffolds 172
GelMA-bp-PRP	3D-bioprinted	promoting osteogenics	bone tissue engineering scaffolds 173
